# A Novel Drug Delivery System for the Treatment of Lupus Nephritis: From Delivery System Design and Optimization to Treatment

**DOI:** 10.3390/biom16030476

**Published:** 2026-03-23

**Authors:** Xumeng Xiong, Jin Tao, Zequn Jin, Ying Hu

**Affiliations:** 1 Cixi Biomedical Research Institute, Wenzhou Medical University, Ningbo 315302, China; xumon@wmu.edu.cn; 2 School of Pharmacy, Zhejiang Pharmaceutical University, Ningbo 315100, China; taoj@zjpu.edu.cn; 3 College of Biological and Environmental Sciences, Zhejiang Wanli University, Ningbo 315100, China; 2023881064@zwu.edu.cn

**Keywords:** lupus nephritis, self-nanoemulsifying drug delivery systems, nanoemulsion, immune regulation, anti-inflammation

## Abstract

Lupus nephritis (LN) is a severe complication of systemic lupus erythematosus (SLE), characterized by immune system disorders and multiple organ damage. Current clinical treatment of LN requires a complex multi-drug combination, which is often associated with severe side effects and low patient compliance. The aim of this study was to design a self-nanoemulsifying drug delivery system (SNEDDS) co-loading total glucosides of Paeonia (TGP) and dihydroartemisinin (DHA) to increase the solubility of the drug as well as achieve synergistic anti-inflammatory and immunomodulatory effects for LN therapy. Network pharmacology, molecular docking and molecular dynamics simulations were employed to predict the core therapeutic targets and related signaling pathways. The SNEDDS co-loaded with TGP and DHA was optimized via central composite design response surface methodology (CCD-RSM). Its physicochemical properties, particle size and the polydispersity index (PDI) of the optimized formulation were characterized. In vivo therapeutic efficacy was evaluated in MRL/lpr mice by measuring disease-related indicators (urinary protein, serum ANA, and anti-ds-DNA) and inflammatory cytokines (TNF-α, IL-6, and IL-1β). Renal tissue pathology was also examined. All data were analyzed by one-way analysis of variance (ANOVA) with *p* < 0.05 considered statistically significant. The core therapeutic targets predicted with high relevance were AKT1, MAPK1, MAPK3, and RELA. The optimized SNEDDS achieved a high loading capacity of 16.11 ± 0.43 mg/g for TGP and 12.79 ± 1.33 mg/g for DHA, with a particle size of (25.84 ± 0.30) nm and PDI of (0.07 ± 0.02). In MRL/lpr mice, SNEDDS treatment significantly reduced urinary protein levels (*p* < 0.01), serum ANA (*p* < 0.01) and anti-ds-DNA titers (*p* < 0.01) compared with the model group. Additionally, the levels of pro-inflammatory cytokines (TNF-α, IL-6, and IL-1β) were markedly decreased (*p* < 0.05), and renal tissue damage was alleviated. Conclusions: The SNEDDS co-loaded TGP and DHA is a promising oral nanotherapeutic strategy for LN, offering synergistic anti-inflammatory and immunomodulatory effects.

## 1. Introduction

Systemic lupus erythematosus (SLE) is an inflammatory autoimmune disease [1]. Lupus nephritis (LN) is a complication of systemic lupus erythematosus (SLE), with approximately 40% of SLE patients diagnosed with LN. The pathological hallmark of LN is the deposition of immune complexes in the glomeruli, triggering complement activation and the release of inflammatory mediators, ultimately leading to renal tissue fibrosis and renal failure [2]. However, there remains an absence of a formulation capable of concurrently regulating the disease through both anti-inflammatory and immunomodulatory mechanisms. The attainment of these effects necessitates the combination of multiple pharmaceutical agents, a practice that often results in suboptimal patient compliance and deleterious adverse effects [3,4,5,6,7,8]. In most of the current studies on traditional Chinese medicine for LN, natural extracts are made into soups and granules, lacking optimization of the dosage form [9,10]. The problem of low oral bioavailability of traditional herbal medicines has not been solved [11].

Total glucosides of Paeonia (TGP) are a series of glycoside compounds extracted from the dried roots of Paeonia lactiflora, a traditional Chinese medicinal plant belonging to the Ranunculaceae family. Paeoniflorin (Pae) has the highest content of TGP, accounting for at least 34.6% [12]. In clinical settings, TGP is employed in the treatment of autoimmune diseases, including systemic lupus erythematosus (SLE), rheumatoid arthritis (RA), and Sjögren’s syndrome (SS). TGP has been demonstrated to possess both anti-inflammatory and immunomodulatory properties. When employed in conjunction with conventional SLE treatment, it has been observed to reduce disease activity, with the therapeutic effect of the combined therapy being superior to that of conventional treatment alone [13]. Furthermore, the administration of TGP has been demonstrated to reduce the recurrence rate and incidence of adverse reactions [14]. TGP can reduce the release of inflammatory mediators by inhibiting the MAPK signaling pathway [15]. However, the precise mechanism through which TGP exerts its effects in the treatment of LN remains to be elucidated. Dihydroartemisinin (DHA) is a metabolite that is derived from the reduction of artemisinin. In addition to its antimalarial properties, DHA has been shown to possess anti-inflammatory and immunomodulatory effects [16,17,18]. DHA significantly inhibited Th17 cell differentiation while inducing Treg cell differentiation in vitro, decreased RORγt transcription and increased Foxp3, as well as IL-17 and TGF-β levels in lymphocytes, and attenuated SLE symptoms by restoring the Treg/Th17 balance [16]. Despite its limited water solubility, DHA shows great promise as a therapeutic agent for LN. Our research group previously prepared TAT-CLs-DHA/siRNA, which has been demonstrated to treat SLE by inhibiting B cell activation and differentiation through the HMGB1/TLR4/NF-κB pathway [19]. However, the utilization of lipid-based gene drug delivery systems is encumbered by their suboptimal stability, which can potentially lead to toxicity and immune reactions [20]. Therefore, the objective of this study is to design a stable, low-toxicity formulation containing two active components from traditional Chinese medicine.

Self-nanoemulsifying drug delivery systems (SNEDDSs) are anhydrous, homogeneous liquid mixtures composed of an oil phase, surfactants, drugs, and co-emulsifiers or solubilizers. When diluted with water under gentle stirring, they spontaneously form water-in-oil nanoemulsions [21]. Due to their small particle size and high viscosity, nanoemulsions are less prone to aggregation and flocculation, enhancing the solubility of poorly soluble drugs. Nanoemulsion with particle sizes of 10–100 nm can be absorbed more rapidly by lymphatic vessels [22]. Furthermore, nanoemulsions exhibit reduced or even no toxicity in multi-species toxicity tests [23]. In vivo, the stirring required to form SNEDDSs is provided by gastrointestinal peristalsis. SNEDDSs containing diindolylmethane exhibited significantly higher inhibition of breast tumor growth, progression, and lung metastasis in a murine mammary tumor model [24]. Co-delivery of TGP and P-glycoprotein inhibitor nobiletin in SNEDDSs could enhance TGP’s anti-arthritic effects by suppressing P-glycoprotein function and expression [25]. To date, SNEDDS formulations such as sandimmune (cyclosporine), fortovase (saquinavir) and norvir (ritonavir) are available on the market, demonstrating the commercial viability and clinical applicability of SNEDDSs [26]. Following the preparation of the lipophilic DHA and poorly orally bioavailable TGP into an anhydrous homogeneous liquid mixture, in vivo self-emulsification can significantly improve drug absorption in the body.

Overall, this study proposes to design an orally available SNEDDS co-loaded with TGP and DHA, which can achieve anti-inflammatory–immunomodulatory synergism during treatment and improve drug solubility while improving patient compliance.

## 2. Methods

### 2.1. Materials

TGP was obtained from Ningbo Lihua Pharmaceutical Co., Ltd. (Ningbo, China). Paeoniflorin was purchased from Shanghai Yuanye Biotechnology Co., Ltd. (Shanghai, China). DHA was purchased from Shanghai Yuanye Biotechnology Co., Ltd. (Shanghai, China). Peceol™ (Type40), Maisine^®^ CC (GM), and Labrafac™ Lipophile WL1349 (MCT) of Gattefossé (Lyon, France) were provided by Guangzhou Standard Pharma Ltd. (Guangzhou, China). Olive Oil was purchased from Shanghai Reagent General Factory (Shanghai, China). Tween 80 (T80) was purchased from Hunan Erkang Pharmaceutical Group Co., Ltd. (Changsha, China). Kolliphor^®^ RH40 (RH40) and Kolliphor^®^ EL (EL35) were obtained from BASF (Shanghai, China). 1,2-propanediol (PG), glycerol (GL), and polyethylene glycol 400 (PEG400) were purchased from Jiangxi Yipusheng Pharmaceutical Co., Ltd. (Ji’an, China). Absolute ethanol (EtOH) was obtained from Hangzhou Gaojing Fine Chemical Co., Ltd. (Hangzhou, China). Lipopolysaccharides (LPSs) were obtained from SIGMA-ALDRICH (Louis, MO, USA). DMEM basic, F-12 basic and Fetal Bovine Serum (FBS) were purchased from Gibco (USA). Mouse Glomerular Mesangial Cells (SV40-MES-13) and Raw 264.7 were purchased from the National Collection of Authenticated Cell Cultures (Shanghai, China). The CCK-8 assay was obtained from UElandy (Suzhou, China). Prednisolone acetate (PNS) was obtained from Zhejiang Xianju Pharmaceutical Co., Ltd. (Taizhou, China). Urinary creatinine, urinary protein, serum creatinine, serum urea nitrogen, and serum albumin biochemical assay kits were purchased from Nanjing Jiancheng Bioengineering Institute (Nanjing, China). Serum anti-double-stranded DNA antibodies and serum anti-nuclear antibodie enzyme-linked immunosorbent assay (ELISA) kits were obtained from MLBio (Shanghai, China). Serum IL-10 and TNF-α ELISA kits were purchased from Quanzhou Ruixin Biotechnology Co., Ltd. (Quanzhou, China). The Mouse Spleen Lymphocyte Isolation Kit was obtained from Solarbio (Beijing, China). The Fc receptor blocker was obtained from Absin (Shanghai, China). PerCP-Cy5.5 Hamster Anti-Mouse CD3e, BV421 Rat Anti-Mouse CD4 and PE Rat Anti-Mouse Foxp3 were purchased from BD (San Jose, CA, USA). Ultrapure water for all experiments was prepared on Merck Simplicity^®^ UV (Darmstadt, Germany).

### 2.2. Network Pharmacology

#### 2.2.1. Acquisition of Drug-Disease-Associated Targets

Bioactive constituents of TGP were identified using the TCMSP database (https://www.tcmsp-e.com/load_intro.php?id=43, accessed on 10 June 2024) [27,28]. Target proteins associated with active ingredients in TGP were subsequently retrieved from SwissTargetPrediction (http://swisstargetprediction.ch, accessed on 15 June 2024) [29,30,31], GeneCards (https://www.genecards.org/, accessed on 15 June 2024) [32], and TCMSP. The results from these databases were consolidated into a union set, with redundant entries systematically removed to generate a non-redundant dataset of target proteins. Therapeutic targets for LN were retrieved from the GeneCards database and TTD database (https://db.idrblab.net/ttd/, accessed on 15 June 2024) [33] using “systemic lupus erythematosus” and “lupus nephritis” as search terms.

#### 2.2.2. Protein–Protein Interaction (PPI) Network Construction

The intersection of drug targets and LN targets was identified using Venny2.1.0 (accessed on 23 June 2024) [34], with a Venn diagram generated to visualize this overlap. The intersecting targets were imported into String12.0 (https://cn.string-db.org/, accessed on 23 June 2024) [35] to generate the TGP-DHA-LN protein–protein interaction (PPI) network and component–target–pathway network. This PPI network file was subjected to interaction assessment using the MCODE plugin in CytoScape3.9.1 [36], yielding core targets of the TGP-DHA-LN PPI network.

#### 2.2.3. Pathway and Functional Enrichment Analysis

Functional enrichment analysis of gene ontology (GO) and Kyoto Encyclopedia of Genes and Genomes (KEGG) pathways was performed using the STRING 12.0. A TGP-DHA-LN target–pathway network was constructed using Cytoscape 3.9.1. The built-in CytoNCA plugin was employed to analyze network topological parameters—including degree centrality (DC), betweenness centrality (BC), and closeness centrality (CC)—for active ingredients, targets, and signaling pathways. Core targets, critical signaling pathways, and primary pharmacodynamically active components were identified based on these topological parameters.

#### 2.2.4. Molecular Docking Validation

We used AutoDock 4.2.6 to identify the optimal binding conformations between proteins and ligands. PDB files of key targets identified through network pharmacology screening were retrieved from the Protein Data Bank (PDB; https://www.rcsb.org) and cross-validated against UniProt (https://www.uniprot.org) [37] for target protein accuracy. SDF files of active ingredients were obtained from PubChem (https://pubchem.ncbi.nlm.nih.gov) [38]. AutoDock Tools [39] was employed to predict docking sites between protein active ingredients. Molecular docking results with binding energies ≤ −5 kcal/mol were considered viable, and optimal binding sites were selected for visualization using PyMOL 2.6.0.

### 2.3. Formulation and Characterization of SNEDDS

#### 2.3.1. HPLC Analytical Method

Paeoniflorin and dihydroartemisinin were analyzed using an Agilent Technologies 1260 Infinity HPLC system (Agilent, Waldbronn, Germerny) with a GL Sciences WondaSil C18 column (4.6 × 250 mm, 5 μm, Shanghai, China). Since paeoniflorin is the most abundant active component in TGP, the solubility of TGP in subsequent evaluations was extrapolated from the quantified paeoniflorin content. Based on our team’s previous experiments, the paeoniflorin content in the TGP used in this study was 36.5%. For paeoniflorin analysis, the mobile phase consisted of 20% acetonitrile and 80% water, with a total run time of 20 min. The detection wavelength was set at 230 nm, column temperature maintained at 30 °C, flow rate at 1.0 mL/min, and injection volume at 10 μL. For dihydroartemisinin analysis, the mobile phase was composed of 60% acetonitrile and 40% water, with a 20 min run time. The detection wavelength was 216 nm, column temperature was 30 °C, flow rate was 1.0 mL/min, and the injection volume was 20 μL.

#### 2.3.2. Equilibrium Solubility Test

Equilibrium solubility studies were conducted using the shake-flask method. Excess amounts of TGP and DHA bulk drugs were added to test tubes, followed by the addition of 1 g each of the oil phases (Type40, GM, MCT and OO), emulsifiers (T80, RH40 and EL35) and co-emulsifiers (PG, EtOH, GL and PEG400). The mixtures were ultrasonically dissolved for 20 min, vortexed to homogeneity, and then shaken at 50 rpm/min for 24 h maintained at 37 °C. After centrifugation at 12,000 rpm/min for 10 min, the supernatants were diluted with methanol and filtered. The solubility of DHA and TGP in different excipients was determined using chromatographic conditions.

#### 2.3.3. Self-Emulsifying Grade Classification

The self-emulsification process is categorized into five grades:

- A. Rapid dispersion and emulsification, forming a clear or slightly bluish microemulsion.
- B. Rapid dispersion and emulsification, forming a blue-white microemulsion.
- C. Slightly slower dispersion and emulsification, forming a bright white milky emulsion.
- D. Slower dispersion and emulsification, with the liquid appearing dark grayish-white and slightly oily in appearance.
- E. Difficult dispersion and emulsification, unable to form a uniform system, with oil droplets persisting throughout.

Among the five grades, Grade A exhibits the best self-emulsifying effect, while Grade E shows the poorest self-emulsifying effect, and so on.

#### 2.3.4. Evaluation of Compatibility Between Oil and Emulsifiers

We mixed the oil phase with emulsifiers that have better drug solubility (RH40, T80, EL35) at ratios of 1:2, 1:1.5, 1:1, 1.5:1, and 2:1 at 37 °C until thoroughly blended. We added 100 times the volume of ultrapure water and gently stirred with a magnetic stirrer to observe the emulsification process. For the evaluation of the emulsification results, refer to Section 2.3.3.

#### 2.3.5. Selection of Co-Emulsifiers

Co-emulsifiers were selected by drawing a pseudo-ternary phase diagram using the non-water drop method. Based on the results of the previous solubility evaluation, Type 40 and MCT were fixed as the oil phase, and PEG400 and EtOH, which both exhibit excellent solubility performance for the two drugs, were selected for further screening. The emulsifier and co-emulsifier were first mixed at different mass ratios (KM) of 1:9, 2:8, 3:7, 4:6, 5:5, 6:4, 7:3, 8:2, and 9:1, and then mixed with the oil phase in the same ratios of 1:9, 2:8, 3:7, 4:6, 5:5, 6:4, 7:3, 8:2, and 9:1. The total system was fixed at 1 g, vortexed to homogenize, diluted 100-fold with 37 °C ultrapure water, gently stirred under magnetic stirring, and the emulsification phenomenon was observed and evaluated. The masses of the oil phase, emulsifier, and co-emulsifier were recorded and used as the vertices of a ternary phase diagram. Using Origin9, the formulation evaluated as A was plotted as a pseudo-ternary phase diagram, and the emulsifier with the largest emulsified area was selected as the emulsifier for subsequent assessment.

#### 2.3.6. Prescription Optimization and Determination of Drug Loading Capacity

Based on the effective microemulsion region, Design Expert 10 was used to optimize the SNEDDS prescription using a 2-factor 5-level central composite design (CCD) response surface methodology (CCD-RSM) to optimize the SNEDDS formulation. Two factors significantly affecting particle size—oil phase mass fraction (X_1_) and the mass ratio of emulsifier to co-emulsifier (KM, X_2_)—were selected as independent variables, while the particle size of self-emulsified droplets (Y_1_) and PDI (Y_2_) were used as dependent variables. Thirteen experiments were conducted according to the design, and the response surface plots were plotted using Origin9. After screening for the optimal formulation, an excess amount of drug was added to the SNEDDS. The supernatant was then collected via centrifugation and sedimentation. The drug loading capacity, particle size, and PDI of the optimal formulation were measured. The drug loading capacity is calculated as follows:

(1) Drug loading capacity (mg/g) = Drug weight (mg)/Gross weight (g) × 100%,

#### 2.3.7. Stability Evaluation

We evaluated the emulsion types of the SNEDDS after emulsification and the effects of different solvents on emulsification outcomes. The evaluation methods for emulsion types are in Section 2.3.3. Different solvents included different temperatures, dilution ratios, and pH levels. We prepared 0.5 g of the SNEDDS according to the optimal formulation ratio. We added the SNEDDS to water/phosphate buffer solutions at different temperatures (4, 25, 37, and 50 °C), different dilution factors (5, 10, 25, 50, and 100 mL), or different pH levels (4.00, 6.86, 7.40, and 9.18), and then mixed uniformly at a constant speed using a magnetic stirrer. W measured the particle size and PDI to examine the self-emulsification.

#### 2.3.8. In Vitro Release Assessment

We simulated in vitro release using the dialysis bag method. A 500 mL, pH 1.2, 0.1 M HCl solution was used as the release medium to simulate gastric fluid conditions, while a pH 6.8 phosphate buffer solution simulated the small intestinal environment. The 1 g SNEDDS co-loaded with 15 mg TGP and 12 mg DHA was placed in a 3500 MW dialysis bag and immersed in different release media at 37 °C. The mixture was stirred at 50 r/min, and samples were collected at specific time points for analysis. This approach evaluated the in vitro release behavior of the SNEDDS under various physiological conditions.

### 2.4. Therapeutic Efficacy Evaluation In Vitro

#### 2.4.1. Cell Culture

DMEM medium, F12 medium, 10% FBS, 100 μm/mL streptomycin and 100 U/mL penicillin were used for SV40-MES-13 growth. DMEM medium and 10% FBS were used for Raw264.7 growth. Cells were maintained at 37 °C in a humidified incubator with 5% CO_2_.

#### 2.4.2. Cell Proliferation Inhibition Assessment

The effects of LPSs at concentrations of 0, 1, 5, 10, 25, and 50 μg/mL on the proliferation of SV40-MES-13 cells were evaluated. SV40-MES-13 cells were seeded into 96-well plates at a density of 5 × 10^4^ cells/mL and incubated overnight at 37 °C. Cells were stimulated with LPS at different concentrations for 5 h. The CCK-8 assay kit was used to evaluate cell proliferation and determine the optimal LPS concentration for stimulating proliferation. The cell population was subjected to administration of the drug to assess the inhibitory effect of the drug on the proliferation of SV40-MES-13 cells under LPS stimulation for 5 h. The cells were grouped as follows:

- Control group: Complete medium.
- Model group: Complete medium with 10 μg/mL LPS.
- TGP SNEDDS group: Complete medium with 10 μg/mL LPS and 0.16 μg/mL TGP SNEDDS.
- DHA SNEDDS group: Complete medium with 10 μg/mL LPS and 0.1 μg/mL DHA SNEDDS.
- Suspension group: Complete medium with 10 μg/mL LPS, 0.16 μg/mL TGP and 0.1 μg/mL DHA.
- TGP + DHA SNEDDS group: Complete medium with 10 μg/mL LPS, 0.16 μg/mL TGP and 0.1 μg/mL DHA SNEDDS.

Following treatment, 10 μL of CCK-8 solution was added to each well and incubated in the dark for 2 h. Absorbance was measured at 450 nm using a microplate reader. Cell viability was calculated according to the following formula (blank contains only medium):

(2) Cell viability (%) = (OD_treated_ − OD_blank_)/(OD_control_ − OD_blank_) × 100%,

#### 2.4.3. Evaluation of Anti-Inflammatory Effects in Raw264.7 Cells

Different concentrations of LPS, 0, 0.1, 0.5, 5, 10, and 15 μg/mL, were used to evaluate the pro-inflammatory effects of Raw264.7 cells. Raw264.7 cells were seeded into 24-well plates at a density of 5 × 10^4^ cells/mL and incubated overnight at 37 °C. Cells were stimulated with LPSs at different concentrations for 12 h. RNA was extracted from the stimulated cells. PT-qPCR experiments were performed to determine the expression of inflammatory factors and to screen for optimal LPS stimulation concentrations. The primer sequences (5′-3′) are as follows:

- GAPDH F: CAGGAGAGTGTTTCCTCGTCC.
- GAPDH R: GATGGGCTTCCCGTTGATGA.
- TNF-α F: CACGCTCTTCTGTCTACTGAACTTC.
- TNF-α R: CTTGGTGGTTTGTGAGTGTGAGG.
- IL-6 F: CAACGATGATGCACTTGCAGA.
- IL-6 R: TGACTCCAGCTTATCTCTTGGT.

After 12 h of treatment with the screened optimal LPS stimulation of Raw264.7 cells along with group drug administration, Western blotting and an RT-qPCR assay were used to assess the therapeutic effect. The grouping was consistent with Section 2.4.2, except that the stimulating concentration of LPSs was 5 μg/mL.

### 2.5. Therapeutic Efficacy Evaluation In Vivo

#### 2.5.1. Laboratory Animals and Treatment Regimen

Six-week-old C57BL/6 and MRL/lpr mice were purchased from Shanghai SLAC Laboratory Animal Co., Ltd. (Shanghai, China). The animals were housed in the SPF animal facility at the Shanghai Institute of Materia Medica, maintained at a temperature of 22 ± 1 °C, humidity of 60–80%, and a 12 h light–dark cycle. Feed and water were provided in disinfected containers. All experiments were conducted in strict accordance with relevant regulations for animal experimentation.

Laboratory mice (all mice except the control group were MRL/lpr mice) were arranged as follows:

- Control group: Saline solution orally.
- Model group: Saline solution orally.
- Positive group: 4.55 mg/kg/day of PNS administered orally.
- Suspension group: 75 mg/kg/day of TGP + 45 mg/kg/day of DHA suspension administered orally.
- SNEDDS group: 75 mg/kg/day TGP + 45 mg/kg/day DHA SNEDDS administered orally.

Mice were treated starting from the eighth week and continued for 10 weeks, with concurrent observation of skin and hair loss.

#### 2.5.2. Skin Damage and Organ Index Assessment

MRL/lpr mice exhibit varying degrees of skin damage and submandibular lymph node swelling as they age, with severe skin lesions appearing at 16–18 weeks of age [40], such as hair loss on the face accompanied by skin bleeding and scabbing. At the end of the experiment, the mice were scored according to the following criteria:

- 1 point = Almost no hair loss around the mouth.
- 2 points = Mild hair loss observed around the mouth.
- 3–4 points = Moderate hair loss observed around the mouth, face, and neck.
- 5–6 points = Severe skin lesions observed on the face, ears, and back of the neck.
- 7–8 points = Very severe rashes and scabbing observed on the face, ears, and back of the neck.

Increased disease activity is often accompanied by lymphoid hyperplasia and splenomegaly [41], so we weighed the collected submandibular lymph nodes and spleen and assessed their organ index (*n* ≥ 4), calculated as follows:

(3) Organ index = Organ weight (g)/Body weight (g).

#### 2.5.3. Pathological Analysis of Kidney H&E and Masson Staining

The vertically sectioned mouse kidneys were fixed, dehydrated, and clarified, then embedded in paraffin and sectioned. The prepared paraffin sections were stained with H&E and Masson staining and finally sealed and stored. The stained kidney sections were photographed, and images were collected using an inverted biological microscope (Wetzlar, Germany), and the images were analyzed.

#### 2.5.4. Serum and Urine Marker Assays

At the end of the experiment, we collected serum and urine samples from each group (*n* ≥ 4) and measured the urinary protein, serum creatinine, urinary creatinine, serum albumin, serum urea nitrogen, serum anti-double-stranded DNA, serum ANA, IL-6, and TNF-α. Procedures were performed according to the kit instructions, and data were statistically analyzed after measuring the absorbance values using a microplate reader (Waltham, MA, USA).

#### 2.5.5. Flow Cytometry Analysis

To explore the therapeutic mechanism of the drug for SLE, we extracted lymphocytes (*n* = 3) from the spleens and submandibular lymph nodes of mice in each group and analyzed cytokine levels using flow cytometry. Spleens were ground in pre-chilled buffer and filtered, then processed according to the instructions of the Mouse Spleen Lymphocyte Isolation Kit (Beijing, China). After collecting the lymphocytes, Fc receptor blocker was added at 4 °C to prevent non-specific binding. Cells were washed with Staining Buffer, resuspended, and incubated at 4 °C with PerCP-Cy5.5 Hamster Anti-Mouse CD3e and BV421 Rat Anti-Mouse CD4 at 4 °C in the dark for 30 min. Then, we added Lysing Buffer, Alexa Fluor^®^ 647 Rat Anti-Mouse IL-17A, PE Rat Anti-Mouse Foxp3, and FITC Rat Anti-Mouse IFN-γ to lyse the cells at 4 °C. After incubation in the dark, we resuspended the cells, filtered them, and used the CytoFlex S instrument (Brea, California, USA) and CytExpert 2.0 software to detect and analyze the expression levels of IFN-γ, IL-17A, and Foxp3 in CD3^+^CD4^+^ T cells from each group of mice. We processed and analyzed the results using FlowJo v10.8.1.

### 2.6. Statistical Analysis

Data were obtained from at least three independent experiments. Statistical significances of the differences in multiple groups were analyzed by one-way ANOVA using GraphPad Prism 8.

## 3. Results

### 3.1. Network Pharmacology, Molecular Docking and Molecular Dynamics Simulations

#### 3.1.1. Screening of Drug-Disease-Related Targets

**Figure 1:**
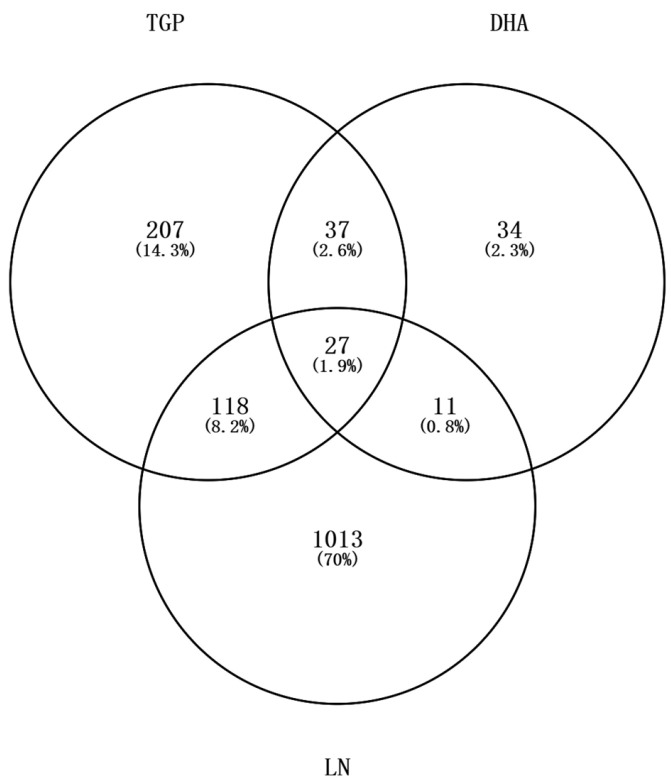
Schematic diagram of target intersection between TGP-DHA and LN (generated by Venny 2.1.0).

After ADME screening, 13 active components of TGP were identified (Table A1). Among these, 11 had related targets, one was a duplicate value, and one had no related targets identified. Finally, after removing duplicates, 390 TGP targets and 109 DHA targets were obtained. Ultimately, we obtained 1099 targets related to “systemic lupus erythematosus”, 321 targets related to “lupus nephritis”, 251 overlapping targets, and a total of 1169 targets in the union. By plotting Venn diagrams (Figure 1), 156 targets for drug–disease correlation were found.

#### 3.1.2. PPI Network Screening Targets

**Figure 2:**
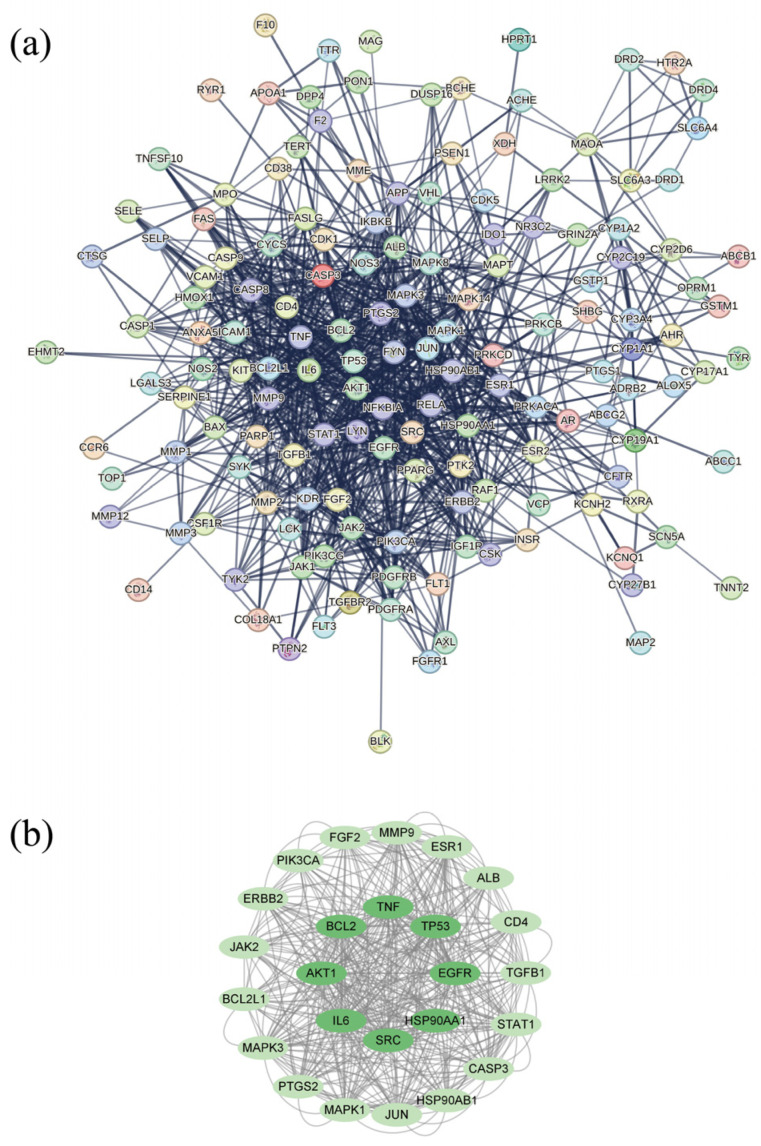
PPI network and core targets of MCODE. (**a**) PPI network of drug–disease targets; (**b**) core PPI targets (dark green indicates the eight core targets with the strongest correlation).

We imported the intersection targets into String12.0 and obtained the drug–disease target PPI network (Figure 2a). The MCODE plugin in CytoScape3.9.1 was used to screen for core PPI targets. The eight most central targets were TP53, IL6, EGFR, SRC, TNF, AKT1, HSP90 AA1, and BCL2 (Figure 2b).

#### 3.1.3. GO, KEGG and Component–Target–Pathway Network

**Figure 3:**
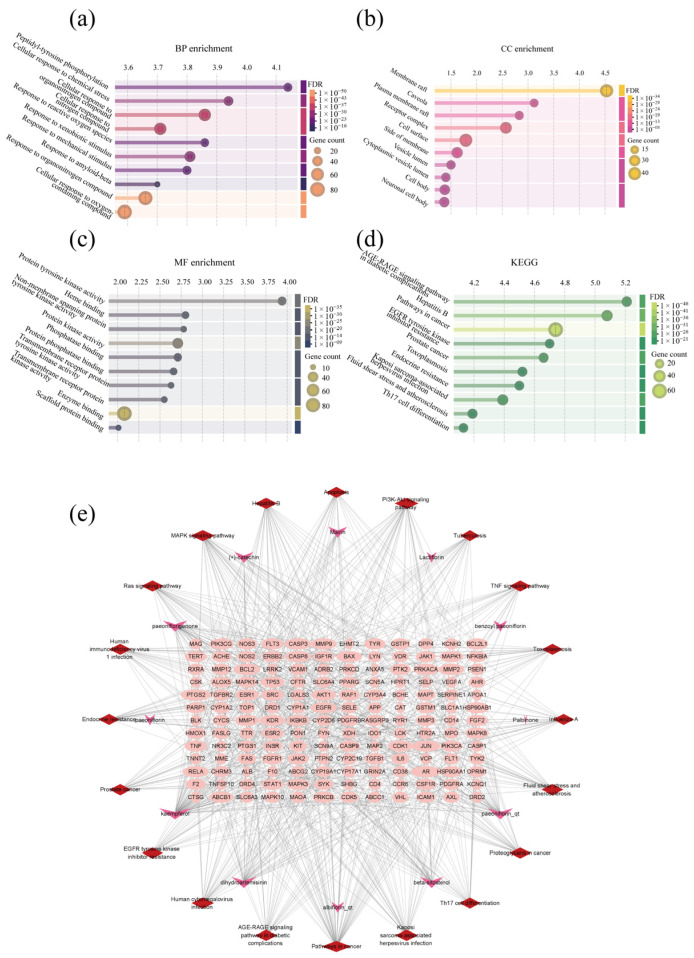
GO, KEGG and component–target–pathway network. (**a**) Top 10 KEGG pathways; (**b**) top 10 biological processes; (**c**) top 10 cellular components; (**d**) top 10 molecular functions; (**e**) TGP-DHA-LN target–pathway network diagram.

The top 10 KEGG pathways (Figure 3a) include the AGE-RAGE signaling pathway in diabetic complications, Hepatitis B, pathways in cancer, EGFR tyrosine kinase inhibitor resistance and so on. The biological process (BP) enrichment (Figure 3b) included peptidyl–tyrosine phosphorylation, cellular response to chemical stress, cellular response to organonitrogen compound, cellular response to nitrogen compound and so on. The cellular component (CC) enrichment (Figure 3c) included a membrane raft, caveola, plasma membrane raft, receptor complex, cell surface, side of membrane, vesicle lumen, cytoplasmic vesicle lumen, cell body and neuronal cell body. The molecular function (MF) enrichment (Figure 3d) included protein tyrosine kinase activity, heme binding, non-membrane spanning protein tyrosine kinase activity, protein kinase activity, phosphatase binding, protein phosphatase binding and so on.

The component–target–pathway network (Figure 3e) was constructed using Cytoscape3.9.1. Node sizes in the network were determined based on closeness centrality, with larger nodes indicating higher connectivity. Red diamonds represent signaling pathways. Plum-colored arrows represent active components. Pink ellipses represent related targets.

#### 3.1.4. Molecular Docking Analysis

**Figure 4:**
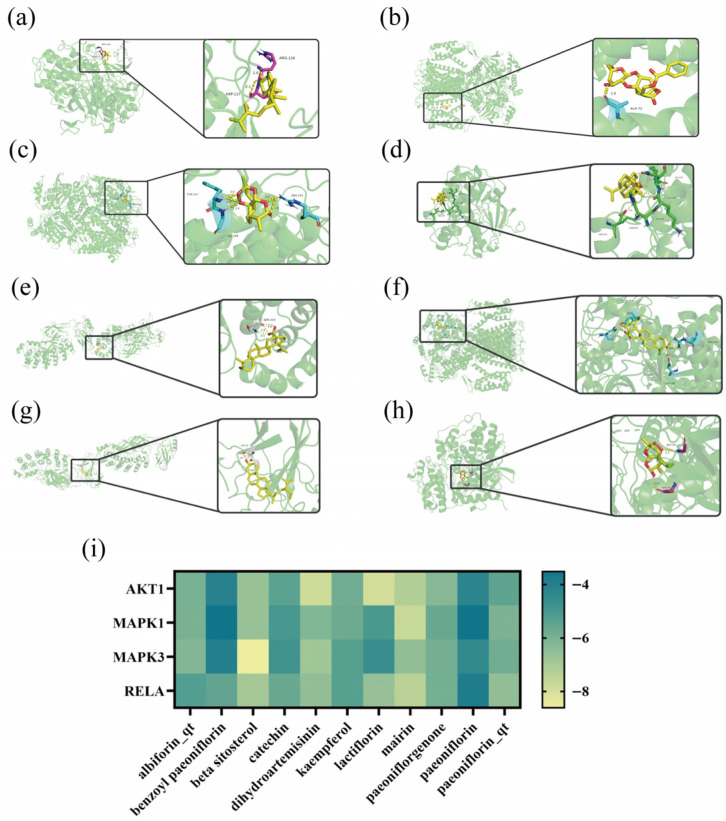
Molecular docking between key components and targets. Yellow dashed lines represent hydrogen bond interactions, and the adjacent numbers indicate bond lengths in Ångströms (Å) (The yellow dashed lines in (**a**–**h**) represent hydrogen bonds between the active ingredient and the target). Clearer original images of the molecular docking can be found in Supplementary Material S1. (**a**) Binding result of MAPK3 and β-sitosterol, binding energy = −8.61 kcal/mol; (**b**) binding result of AKT1 and lactiflorin, binding energy = −7.93 kcal/mol; (**c**) binding result of AKT1 and dihydroartemisinin, binding energy = −7.86 kcal/mol; (**d**) binding result of MAPK1 and mairin, binding energy = −7.74 kcal/mol; (**e**) binding result of RELA and mairin, binding energy = −7.4 kcal/mol; (**f**) binding result of AKT1 and mairin, binding energy = −7.23 kcal/mol; (**g**) binding result of RELA and β-sitosterol, binding energy = −6.92 kcal/mol; (**h**) binding result of MAPK3 and dihydroartemisinin, binding energy = −6.88 kcal/mol; (**i**) heatmap of binding energies between core targets and active components.

Four selected important proteins, MAPK3 (PDB ID: 2zoq), AKT1 (PDB ID: 7fcv), RELA (PDB ID: 1nfi), and MAPK1 (PDB ID: 7E75), were downloaded from the PDB website and performed molecular docking with 11 active ingredients using Auto Dock 4.2.6. Based on the final binding energy calculations, we selected eight results with lower binding energy and visualized the binding outcomes between the proteins and small molecules using PyMOL. The results are shown in Figure 4. The most stable binding is observed with AKT1. β-Sitosterol in TGP exhibits binding energies ranging from −6.65 to −8.61 kcal/mol with MAPK1, MAPK3, RELA, and AKT1. The binding energy with MAPK3 is −8.61 kcal/mol, indicating strong binding. DHA exhibits binding energies ranging from −6.2 to −7.86 kcal/mol with these four proteins. These results further support the potential of TGP and DHA to synergistically treat LN through immune and anti-inflammatory mechanisms.

### 3.2. Formulation and Characterization of SNEDDS

#### 3.2.1. Screening of Excipients for Higher Solubility

**Figure 5:**
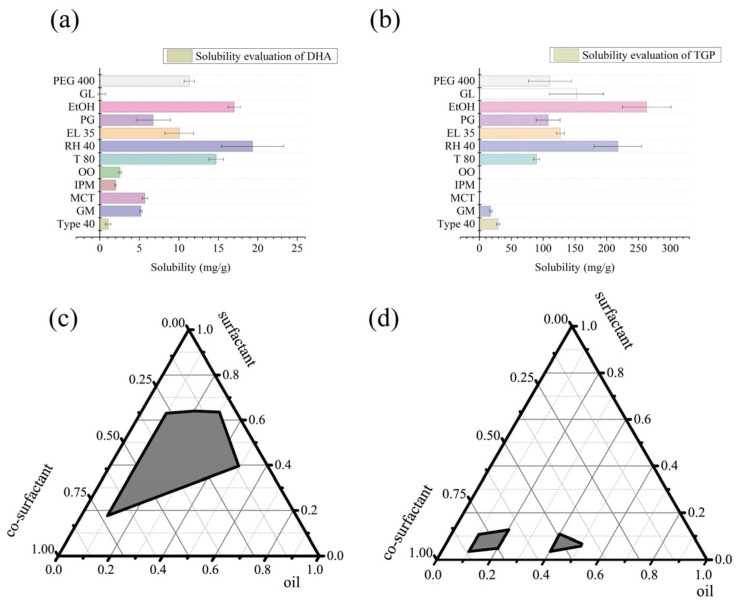
Solubility evaluation of DHA and TGP (*n* = 3) and pseudo-ternary phase diagrams for co-emulsifier screening. (**a**) Solubility of DHA in different excipients; (**b**) solubility of TGP in different excipients. (**c**) Pseudo-ternary phase diagram with Type40 and MCT as the oil phase, RH40 as the emulsifier, and EtOH as the co-emulsifier; (**d**) pseudo-ternary phase diagram with Type40 and MCT as the oil phase, RH40 as the emulsifier, and PEG400 as the co-emulsifier.

The solubility results (Figure 5) of TGP and DHA in different excipients were tested. Based on the solubility screening results, the oil phases Type40, GM, and MCT exhibit superior drug solubility performance. Among these, Type40 and GM are long-chain oils, while MCT is a medium-chain oil. Medium-chain oils and long-chain oils have different characteristics in terms of in vivo absorption: medium-chain oils can promote lymphatic absorption of drugs, while long-chain oils can enhance drug permeability. To enhance drug absorption in the body, based on the solubility screening results, subsequent experiments selected a mixed oil phase (Type40: MCT = 4:3) for formulation screening and optimization.

#### 3.2.2. Evaluation of Compatibility Between Oil and Emulsifiers

The Type40 and MCT oil phases were mixed with three emulsifiers at different ratios at 37 °C until thoroughly blended. The amount of ultrapure water was 100 times the volume of emulsion added, and the emulsification process was observed under gentle magnetic stirring. The emulsification results of the oil phase and emulsifiers at different ratios are shown in Table 1. Compatibility tests indicated that RH40 yielded the most ideal emulsification effect, with a transparent appearance and a blue emulsion sheen. Therefore, RH40 was selected as the emulsifier.

#### 3.2.3. Selection of Co-Emulsifier

The formulation evaluated as “A” for emulsification using Origin9 was used to plot the pseudo-ternary phase diagram. The results show that systems containing PEG400 (Figure 5d) can solubilize more oil phase, while systems containing EtOH (Figure 5c) have a larger emulsified area. However, during the experiment, systems using PEG400 as the co-emulsifier had weak drug-carrying capacity, with a large amount of drug precipitating after self-emulsification. In contrast, systems using EtOH as the co-emulsifier exhibited significantly enhanced drug-carrying capacity. Therefore, EtOH was selected as the co-emulsifier.

#### 3.2.4. Prescription Optimization and Determination of Drug Loading Capacity

A CCD-RSM experiment was designed using Design Expert 10, with the results summarized in Table 2. The experimental data were subjected to multiple linear regression and quadratic polynomial fitting. The fitting function was determined based on the principles that the adjusted correlation coefficient of the equation passed the F-test and the R^2^ value was maximized.

**Figure 6:**
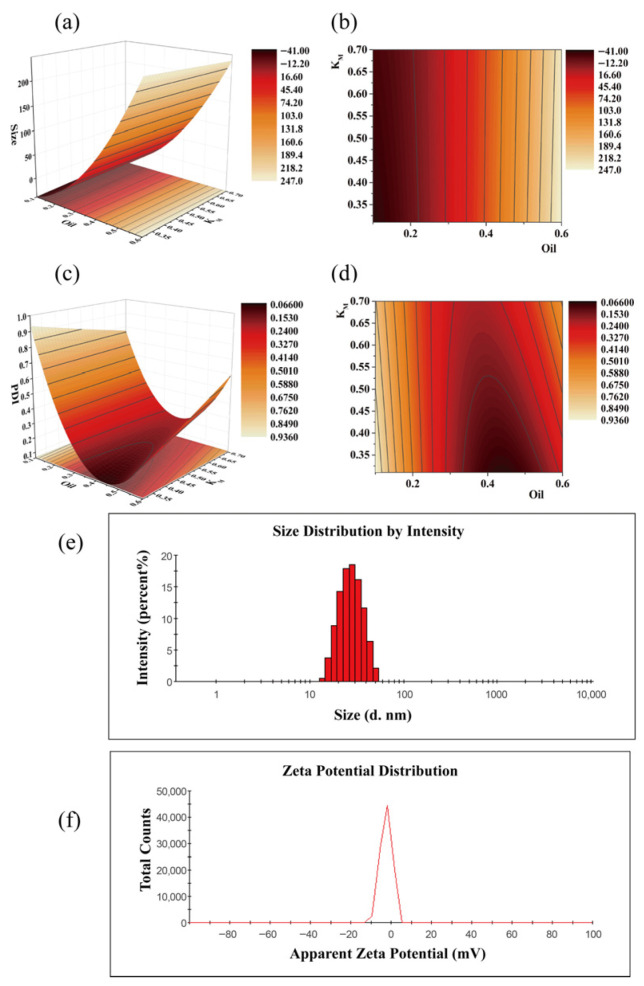
Response surface plots and contour plots of Z-size and PDI based on CCD-RSM experimental design. (**a**) Response surface plot of Z-size; (**b**) contour plots of Z-size; (**c**) response surface plot of PDI; (**d**) contour plots of PDI; (**e**) particle size and PDI of nanoemulsion; (**f**) Zeta potential of nanoemulsion.

The actual fitting equation for particle size is as follows:

(4) Y_1_ = − 54.27253 − 25.56318X_1_ + 35.46811X_2_ − 107.82667X_1_X_2_ + 903.75733X_1_^2^ − 1.05785X_2_^2^, R^2^ = 0.9373,

The actual fitting equation for the PDI is as follows:

(5) Y_2_ = 1.75438 − 7.41525X_1_ − 0.7866X_2_ + 2.57925X_1_X_2_ + 7.52716X_1_^2^ + 0.10664X_2_^2^ − 0.92970X_1_^2^X_2_ − 0.30629X_1_X_2_^2^, R^2^ = 0.9470,

The star-point response surface diagrams (Figure 6a–d) were plotted using Origin 9. Finally, the oil phase constitutes 35% of the SNEDDS, consisting of a blend of Type40 and MCT in a 4:3 ratio; the emulsifier is RH40, accounting for 51%, with ethanol (EtOH) serving as the co-emulsifier at 15%. The optimal formulation can simultaneously accommodate up to 16.11 ± 0.43 mg/g of TGP and 12.79 ± 1.33 mg/g of DHA. After emulsification with water dilution, the nanoemulsion had particle size of 25.84 ± 0.30 nm (Figure 6e), PDI of 0.07 ± 0.02 and Zeta potential of −1.54 ± 0.61 (Figure 6f). The particle size of the nanoemulsion primarily distributed between 18.17 and 32.67 nm (67%), with the highest proportion (18.6%) occurring in the 24.36–28.21 nm range. This indicated uniform particle size distribution after emulsification of the SNEDDS with water.

#### 3.2.5. Stability Evaluation

The SNEDDS exhibited similar emulsification results (Table 3) in different aqueous solutions, with Z-size consistently below 35 nm and the PDI below 0.300. No drug precipitation occurred after one week of storage, demonstrating excellent stability.

#### 3.2.6. In Vitro Release Assessment

**Figure 7:**
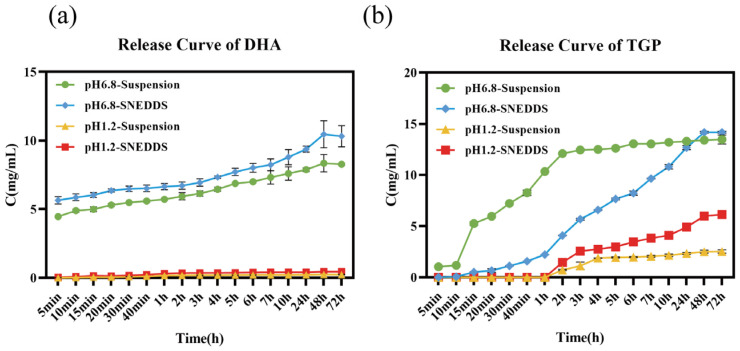
Release profiles of DHA and TGP from mixtures and SNEDDS under different release media (*n* = 3): (**a**) 72 h release profiles of DHA from mixtures and SNEDDS at pH 1.2 and pH 6.8; (**b**) 72 h release profiles of TGP from mixtures and SNEDDS at pH 1.2 and pH 6.8.

Based on the release profiles of TGP and DHA in the suspension and SNEDDS (Figure 7), the cumulative release of DHA and TGP drugs was very limited in both the suspension group and the SNEDDS group at pH 1.2. Drug release behavior in simulated intestinal fluid at pH 6.8 exhibited significant changes. The release rates of TGP and DHA accelerated markedly, with cumulative release quantities increasing steadily over time. Overall, the release rates of the formulation groups were higher than those of the suspension group, indicating that the SNEDDS enhances in vitro drug dissolution and improves in vivo absorption.

### 3.3. Therapeutic Efficacy Evaluation In Vitro

#### 3.3.1. SNEDDS Treatment Inhibits SV40-MES-13 Cell Proliferation Stimulated by LPSs

**Figure 8:**
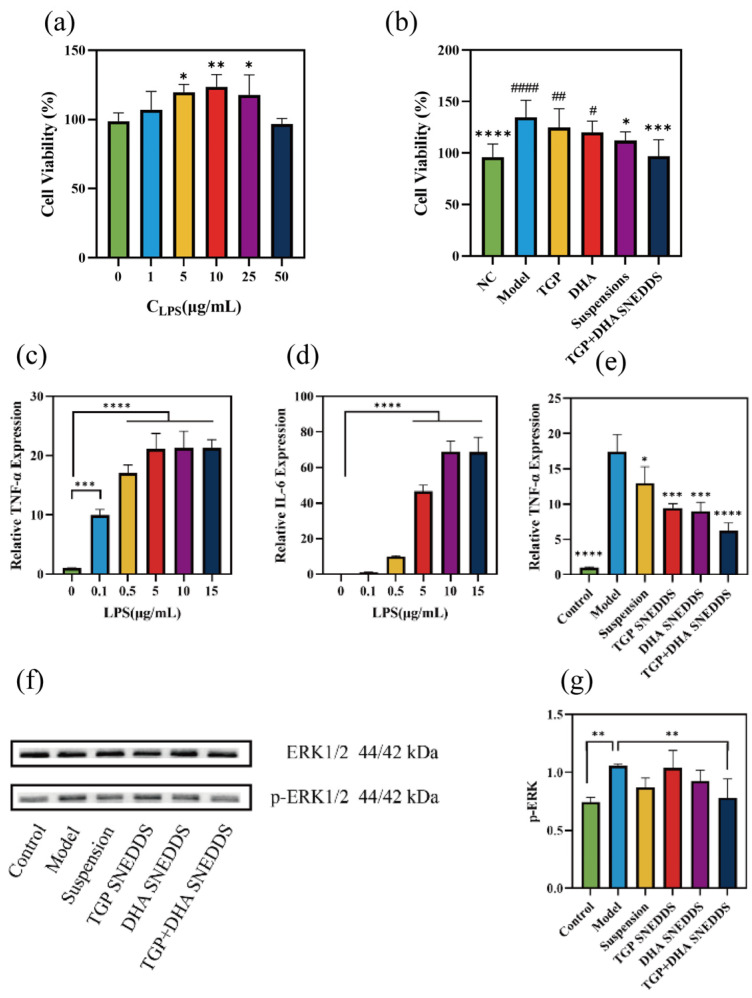
Therapeutic efficacy evaluation in SV40-MES-13 cells (*n* = 6) and in Raw264.7 cells (*n* = 3). (**a**) Proliferative effects of different concentrations of LPSs on SV40-MES-13 cells (compared with blank); (**b**) drug inhibition of SV40-MES-13 cell proliferation caused by LPSs (* compared with model, ^#^ compared with NC). (**c**) Transcriptional levels of TNF-α following stimulation with LPS at different concentrations (* compared with blank); (**d**) transcriptional levels of IL-6 following stimulation with LPS at different concentrations (* compared with model); (**e**) relative TNF-α expression; (**f**) Western blotting was performed for analysis of the p-ERK; Original images can be found in Supplementary Material S2; (**g**) quantitative analysis of p-ERK protein detected by Western blotting.* *p* < 0.05, ** *p* < 0.01, *** *p* < 0.001, **** *p* < 0.0001. ^#^ *p* < 0.05, ^##^ *p* < 0.01, ^####^ *p* < 0.0001.

**Figure 9:**
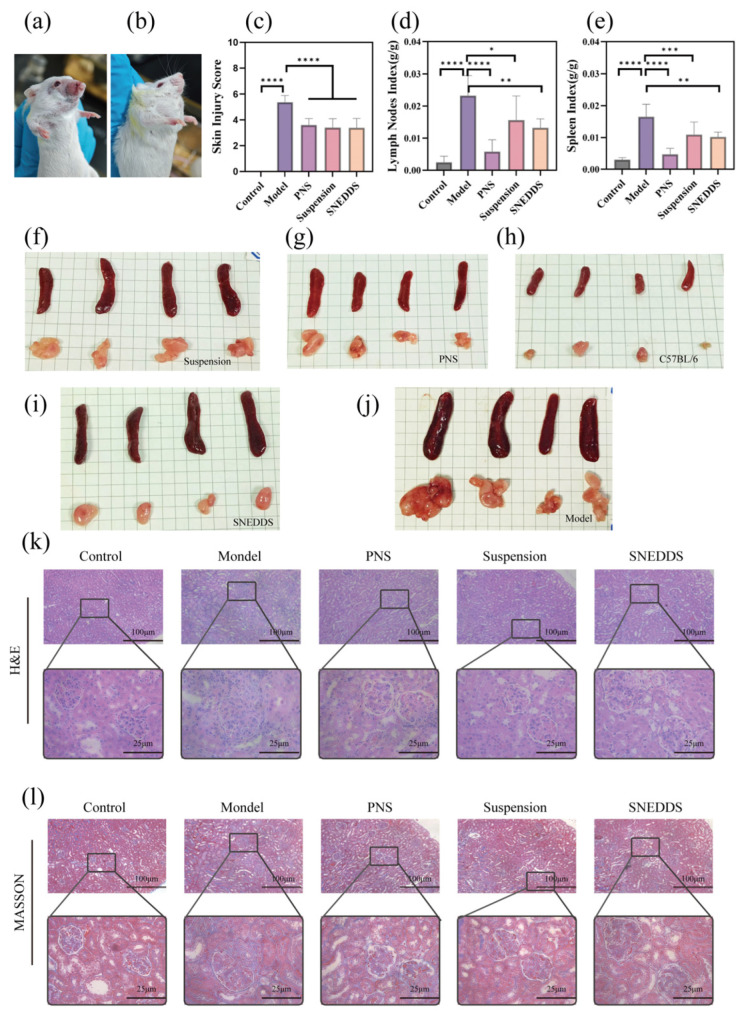
Assessment of skin damage and organ indices in mice (*n* = 4). Histopathological sections of mouse kidneys stained with H&E and Masson’s trichrome stain. (**a**) Skin damage in MRL/lpr mice; (**b**) swelling of the submandibular lymph nodes in MRL/lpr mice; (**c**) assessment of skin damage in mice at week 18; (**d**) assessment of organ index of submandibular lymph nodes in mice at week 18; (**e**) assessment of organ index of spleen in mice at week 18; (**f**–**j**) spleens and submandibular lymph nodes of mice from different groups; (**k**) H&E and stained pathological sections of kidneys from different groups of mice; (**l**) Masson staining of pathological section images of kidneys from different groups of mice. * *p* < 0.05, ** *p* < 0.01, *** *p* < 0.001, **** *p* < 0.0001.

The optimal LPS concentration to stimulate the proliferation of SV40-MES-13 cells was screened by CCK-8 assay. SV40-MES-13 cells stimulated by the administration of 10 μg/mL of LPSs significantly proliferated (Figure 8a) compared to the blank group (*p* < 0.01). Therefore, in the proliferation inhibition evaluation, we stimulated SV40-MES-13 cells with 10 μg/mL of LPSs. Following drug administration, cell proliferation was significantly inhibited in the SNEEDS group compared with the model group (*p* < 0.001), which was not significantly different from the NC group (Figure 9b).

#### 3.3.2. Anti-Inflammatory Effects of SNEDDS in Raw264.7 Cells

The experimental results showed that after stimulation with 5 μg/mL of LPSs, the levels of TNF-α (*p* < 0.0001) and IL-6 (*p* < 0.0001) in Raw264.7 cells were significantly increased compared with those in the blank group (Figure 8c,d). The SNEDDS group showed a significant decrease in TNF-α transcript levels (Figure 8e) after LPS stimulation for 12 h with concomitant drug administration (*p* < 0.0001). The results of Western blotting (Figure 8f,g) showed a decrease in p-ERK expression (*p* < 0.01).

### 3.4. Therapeutic Efficacy Evaluation In Vivo

#### 3.4.1. Skin Damage and Organ Index Assessment

Mice developed skin lesions and submandibular lymph node enlargement at 17 weeks. At week 18, we assessed the skin lesions in the mice and found that the skin lesions in the treated mice were greatly reduced (Figure 9a,b). Based on skin scoring results (Figure 9c), the control group showed no skin damage. The model group exhibited the highest skin damage scores, with severe hair loss on the cheeks and the presence of blood crusts. In contrast, the treated groups—PNS, suspension, and SNEDDS—all demonstrated lower skin damage scores than the model group (*p* < 0.0001), with only minor hair loss observed around the mouth. The lymph nodes of mice in the untreated model group exhibited obvious enlargement compared to the normal group and treatment group. After treatment with PNS and SNEDDS, lymph node hyperplasia and spleen swelling caused by the disease were reduced to varying degrees (Figure 9d,e).

#### 3.4.2. Pathological Analysis of Kidney H&E and Masson Staining

The results of kidney H&E and Masson staining pathological analysis are shown in Figure 9k,l. In the control group, there were no signs of inflammation or damage, indicating normal kidney tissue. The model group exhibited severe lupus nephritis, with glomeruli showing diffuse proliferation and sclerosis, accompanied by thickening of the basement membrane, mesangial proliferation, and inflammatory cell infiltration. The renal tubules exhibited significant cast formation and epithelial necrosis, while the interstitium showed widespread inflammation and fibrosis. In the PNS group, lupus nephritis was partially alleviated, with reduced glomerular proliferation and sclerosis, decreased inflammatory cell infiltration, and attenuated interstitial fibrosis, though mild basement membrane thickening and residual inflammation persisted. In the suspension group, lupus nephritis showed significant improvement after treatment, but mesangial proliferation, segmental sclerosis, and mild interstitial fibrosis remained. In the SNEDDS group, most pathological changes in lupus nephritis improved, with marked relief of glomerular proliferation and sclerosis, basic restoration of tubular structure, and very mild interstitial inflammation and fibrosis.

#### 3.4.3. Disease Marker Testing in Serum and Urine

**Figure 10:**
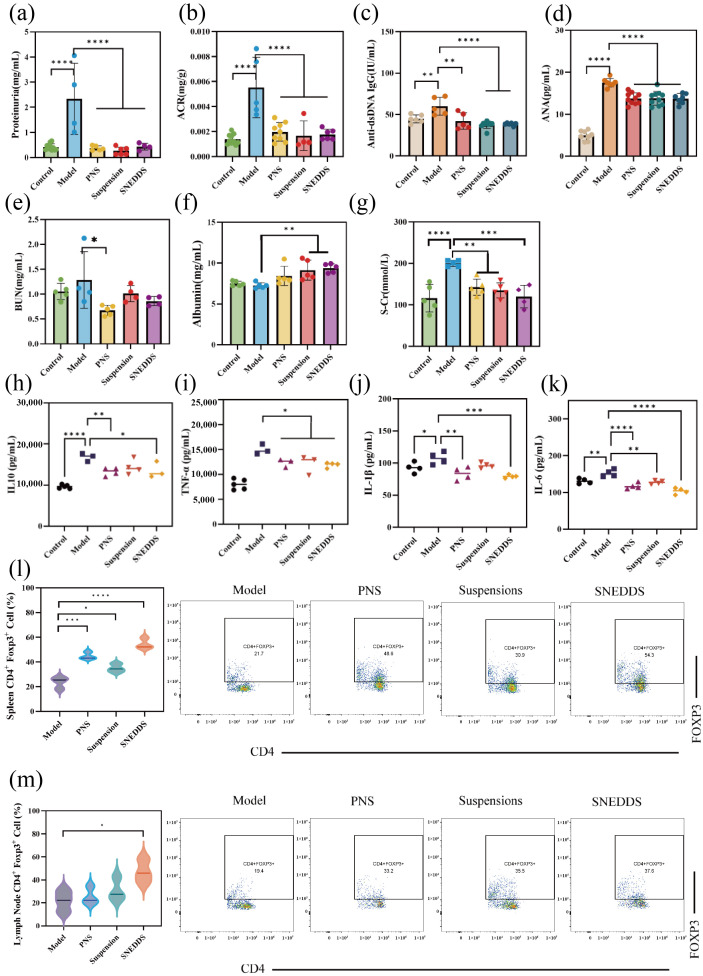
Evaluation of therapeutic efficacy in mice after 10 weeks of administration (*n* ≥ 4). (**a**) Urinary protein levels; (**b**) ACR levels in urine; (**c**) serum anti-ds-DNA IgG levels; (**d**) serum ANA levels; (**e**) serum BUN levels; (**f**) serum ALB levels; (**g**) serum S-Cr levels; (**h**) serum TNF-α levels; (**i**) serum IL-10 levels; (**j**) serum IL-1β levels; (**k**) serum IL-6 levels; (**l**) the expression of CD4^+^Foxp3^+^T cells in spleen; (**m**) the expression of CD4^+^Foxp3^+^T cells lymph nodes. The horizontal line in (**l**,**m**) represents the median. The distribution maps of the flow cytometry detection in Supplementary Material S3. * *p* < 0.05, ** *p* < 0.01, *** *p* < 0.001, **** *p* < 0.0001.

Serum and urine samples were analyzed for various parameters, with the results shown in Figure 10a–k. The urine protein levels in the model group were significantly higher than those in the control group (*p* < 0.0001). Compared with the model group, SNEDDS treatment increased serum ALB concentration (*p* < 0.01), decreased S-Cr levels (*p* < 0.001), and improved renal function in MRL/lpr mice. Among disease detection indicators, SNEDDS treatment also reduced ANA levels (*p* < 0.0001), anti-ds-DNA antibody levels (*p* < 0.0001), IL-10 (*p* < 0.05), TNF-α (*p* < 0.05), IL-1β (*p* < 0.001) and IL-6 (*p* < 0.0001).

#### 3.4.4. Flow Cytometry Detection of Immune Factors

Following treatment (Figure 10l,m), CD4^+^Foxp3^+^ T cell expression in the spleen was significantly higher in the SNEDDS group compared to the model group (*p* < 0.0001). Expression also increased in the PNS group (*p* < 0.001), while the suspension group showed a slight increase in CD4^+^Foxp3^+^ T cells (*p* < 0.05). In lymph nodes, only the suspension group showed a trend toward elevated CD4^+^Foxp3^+^ T cells relative to the model group (*p* < 0.05).

## 4. Discussion

SLE is an inflammatory autoimmune disease characterized by multiple autoimmune phenomena due to an imbalance in the homeostasis of autoreactive lymphocytes, and LN is a serious complication of SLE [1,2]. In this study, we co-loaded TGP and DHA into a novel SNEDDS formulation to achieve co-delivery of the two drugs. Most drugs currently available for treating LN, such as cyclophosphamide and corticosteroids, have only immunomodulatory [3] or anti-inflammatory effects [6]. However, our compound formulation can synergistically treat LN by combining TGP and DHA, while also exerting anti-inflammatory and immunomodulatory effects [7,13,18,42]. Our formulation significantly addresses the issue of poor drug solubility [43]. Although network pharmacology and molecular docking cannot elucidate the mechanism of action between drugs and diseases in vivo, their application has significantly reduced the burden of preliminary drug screening and helped us predict multi-target interactions between drugs and diseases [44]. We utilized network pharmacology to screen for common targets between TGP and DHA with LN through ADME screening and identified 11 active components and key targets—MAPK3, MAPK1, AKT1, and RELA. Then, we used AutoDock 4.2.6 to reveal the interaction mechanisms between receptors and ligands in a computational setting, predicting their binding patterns and affinities [45]. TGP and DHA exhibit good binding properties with MAPK3, MAPK1, AKT1, and RELA. DHA has low solubility, but the SNEDDS we prepared can significantly increase DHA solubility, improve its permeability, and promote absorption in the body [46]. Our SNEDDS can simultaneously encapsulate 15 mg/g TGP and 12 mg/g DHA. After drug loading, the SNEDDS spontaneously forms an O/W emulsion upon water dilution, with a particle size of 25.62 nm and a PDI of 0.102, demonstrating excellent stability. The dialysis bag method [47] was used to evaluate drug release in vitro. In simulated gastric fluid at pH 1.2, the cumulative release of DHA and TGP from both the suspension group and SNEDDS group was extremely limited. This phenomenon may be related to the physicochemical properties of the drugs, as TGP and DHA exhibit low solubility in acidic environments. Furthermore, as a lipophilic drug, DHA may be retained within the oil phase. Drug release behavior markedly changed in simulated intestinal fluid at pH 6.8. The release rates of TGP and DHA accelerated significantly, with cumulative release quantities steadily increasing over time, indicating superior dissolution properties in neutral or weakly alkaline conditions. The formulation group exhibited higher drug release quantities than the suspension group, demonstrating that the SNEDDS enhances in vitro drug dissolution and improves in vivo absorption.

Glomerular mesangial proliferation can also induce LN [48]. Inhibiting the proliferation of glomerular mesangial cells can effectively reduce glomerulosclerosis and alleviate renal lesions [49]. ERK1/2 plays an important role downstream of immune receptors, triggering the expression of inflammatory factors in response to infection and cell or tissue damage [50]. We used SV40-MES-13 cells and Raw264.7 cells to evaluate the therapeutic effects of the SNEDDS. After stimulating SV40-MES-13 cells with 10 µg/mL LPS and administering treatment for 5 h, the drug-loaded SNEDDS exhibited the most pronounced cell proliferation inhibition. Compared to the model group, the SNEDDS group achieved a cell inhibition rate of 37.86%. The cell proliferation inhibition rates for the single-component TGP SNEDDS and DHA SNEDDS were only 10.00% and 14.86%, respectively, indicating that the TGP-DHA SNEDDS exhibits synergistic therapeutic effects. The cell proliferation inhibition rate in the TGP-DHA suspension group was 22.86%, significantly lower than that in the SNEDDS group. The results showed that the SNEDDS inhibited the proliferation of SV40-MES-13 cells and thus alleviated nephritis caused by over-proliferation. The SNEDDS alleviated LPS stimulation-induced inflammation in Raw264.7 cells and reduced ERK phosphorylation. This result, together with network pharmacology and molecular docking results, corroborates the anti-inflammatory effect of the SNEDDS.

To confirm the therapeutic effect of the compound formulation on LN in vivo, we administered the treatment to MRL/lpr mice at 8 weeks of age for a period of 10 weeks. Similar to reports in the literature [51], our MRL/lpr mice exhibited hair loss, skin damage, and even ulcers at 17 and 18 weeks of age. ALB is the most abundant protein in plasma and is excreted renally due to disruption of the filtration barrier during glomerulonephritis. BUN and S-Cr are also critical indicators of renal function, directly reflecting glomerular filtration capacity; their plasma concentrations increase when filtration rates decline [52]. In serum, ALB levels were higher across all groups compared to the model group, while BUN and S-Cr expression decreased, indicating that treatment improved renal function in MRL/lpr mice. SLE patients typically exhibit elevated levels of IL-6, IL-10 and TNF-α [53,54,55]. The disease indicators in the SNEDDS group were significantly lower than those in the model group, indicating that the SNEDDS loaded with TGP and DHA can significantly reduce disease activity. Overall, the PNS and suspension groups demonstrated lower anti-inflammatory efficacy compared to the SNEDDS group. Foxp3 is a specific marker for Treg cells in mice. Clinical studies have shown that the proportion of CD4^+^ Foxp3^+^ T cells in SLE patients is significantly higher than in healthy controls, and disease activity is significantly correlated with the proportion of CD4^+^ Foxp3^+^ T cells [56]. The expression of CD4^+^Foxp3^+^T cells was significantly reduced in the SNEDDS group, while its expressions were reduced only slightly in other groups, suggesting the immunomodulatory effects of the SNEDDS. In vitro and in vivo results indicated that SNEDDSs co-loaded TGP and DHA could synergistically regulate the “Treg cell–inflammation” axis, thereby alleviating renal pathological damage.

## 5. Conclusions

We designed a multi-drug co-loaded SNEDDS with anti-inflammatory and immune synergistic effects. Therapeutic targets were predicted for the disease through network pharmacology and molecular docking; then, we designed an SNEDDS co-loaded with TGP and DHA to enhance drug solubility. The TGP-DHA SNEDDS effectively inhibited LPS-induced proliferation in SV40-MES-13 cells and inflammation in Raw264.7 cells. Therapeutic outcomes in MRL-lpr model mice demonstrated that the TGP-DHA SNEDDS reduced proteinuria, mitigated renal injury, and modulated immune dysfunction to alleviate inflammation. Compared with the complex combination therapy in treatment guidelines, this preparation can greatly improve patient compliance. The synergistic mechanism has implications for the development of LN nanodelivery systems.

## Figures and Tables

**Table 1:** Evaluation of Oil-in-Water Emulsion Compatibility.

Emulsifier	Number	Oil:Emulsifier	Self-Emulsifying Grade
RH40	1	1:2	A
	2	1:1.5	A
	3	1:1	B
	4	1.5:1	C
	5	2:1	D
T80	6	1:2	C
	7	1:1.5	C
	8	1:1	C
	9	1.5:1	C
	10	2:1	D
EL35	11	1:2	A
	12	1:1.5	B
	13	1:1	C
	14	1.5:1	D
	15	2:1	E

**Table 2:** CCD-RSM experiment.

Level	X_1_	X_2_	Z-Size (nm)	PDI
1	0.17 (−1)	2.44 (−1)	18.37	0.103
2	0.35 (0)	2 (−1.414)	28.20	0.098
3	0.1 (−1.414)	3.5 (0)	16.33	0.151
4	0.53 (+1)	4.56 (+1)	42.33	0.217
5	0.35 (0)	3.5 (0)	26.99	0.102
6	0.35 (0)	3.5 (0)	25.93	0.058
7	0.35 (0)	3.5 (0)	25.59	0.058
8	0.35 (0)	5 (+1.414)	26.34	0.093
9	0.35 (0)	3.5 (0)	27.68	0.119
10	0.6 (+1.414)	3.5 (0)	155.93	0.577
11	0.17 (−1)	4.56 (+1)	19.073	0.119
12	0.53 (+1)	2.44 (−1)	122.50	0.363
13	0.35 (0)	3.5 (0)	26.73	0.073

**Table 3:** Stability evaluation (*n* = 3).

Factor		Z-Size (nm)	PDI
Temperature (°C)	4	17.7 ± 0.22	0.046 ± 0.03
	25	25.66 ± 0.08	0.096 ± 0.006
	37	30.21 ± 1.3	0.166 ± 0.024
	50	28.52 ± 1.24	0.257 ± 0.052
Dilution ratio	10	29.27 ± 0.4	0.105 ± 0.039
	20	29.19 ± 0.38	0.133 ± 0.018
	50	30.85 ± 1.16	0.151 ± 0.079
	100	32.83 ± 0.96	0.276 ± 0.023
	200	31.83 ± 0.73	0.252 ± 0.026
pH	4	27.48 ± 1.48	0.064 ± 0.005
	6.86	28.69 ± 2.03	0.094 ± 0.012
	7.4	27.16 ± 2.3	0.066 ± 0.016
	9.18	28.32 ± 1.56	0.065 ± 0.003

## Data Availability

The original contributions presented in this study are included in the article/Supplementary Material. Further inquiries can be directed to the corresponding author.

## Supplementary Materials

The following supporting information can be downloaded at: https://www.mdpi.com/article/10.3390/biom16030476/s1. S1: The original images of the molecular docking; S2: the original images of the Western Blot; S3: the distribution maps of the flow cytometry detection.

## Abbreviations

The following abbreviations are used in this manuscript:

LN	Lupus nephritis
SLE	Systemic lupus erythematosus
SNEDDS	Self-nanoemulsifying drug delivery system
TGP	Total glucosides of Paeonia
DHA	Dihydroartemisinin
CCD-RSM	Central composite design response surface methodology
PDI	Polydispersity index
ANOVA	One-way analysis of variance
RA	Rheumatoid arthritis
SS	Sjögren’s syndrome
Type40	Peceol™
GM	Maisine^®^ CC
MCT	Labrafac™ Lipophile WL1349
OO	Olive Oil
T80	Tween 80
RH40	Kolliphor^®^ RH40
EL35	Kolliphor^®^ EL
PG	1,2-propanediol
GL	Glycerol
PEG400	Polyethylene glycol 400
EtOH	Ethanol
LPS	Lipopolysaccharide

## Appendix A

### Appendix A.1

**Table A1:** Active components of TGP and DHA.

MOL ID	Ingredient	OB(%)	DL	CAS	Number of Targets
MOL001910	BW45NB63RE	64.77	0.38	186140-36-3	0
MOL001918	Paeoniflorigenone	87.59	0.37	80454-42-8	103
MOL001919	Palbinone	43.56	0.53	139954-00-0	4
MOL001921	Lactiflorin	49.12	0.8	1361049-59-3	4
MOL001924	Paeoniflorin	53.87	0.79	23180-57-6	4
MOL001925	Paeoniflorin_qt	68.18	0.4		111
MOL001928	Albiflorin_qt	66.64	0.33		4
MOL001930	Benzoyl paeoniflorin	31.27	0.75	38642-49-8	3
MOL000211	Mairin	55.38	0.78	472-15-1	32
MOL000358	Beta-sitosterol	36.91	0.75	83-46-5	60
MOL000359	Sitosterol	36.91	0.75	83-46-6	60
MOL000422	Kaempferol	41.88	0.24	520-18-3	154
MOL000492	(+)-catechin	54.83	0.24	154-23-4	40
MOL007425	Dihydroartemisinin	50.75	0.30	81496-82-4	98
