# Supplementary material for: A Novel Drug Delivery System for the Treatment of Lupus Nephritis: From Delivery System Design and Optimization to Treatment
Source: Biomolecules. 2026 Mar 23;16(3):476. doi: 10.3390/biom16030476 (PMC13024118; doi:10.3390/biom16030476)

a. Binding result of MAPK3 and β-sitosterol

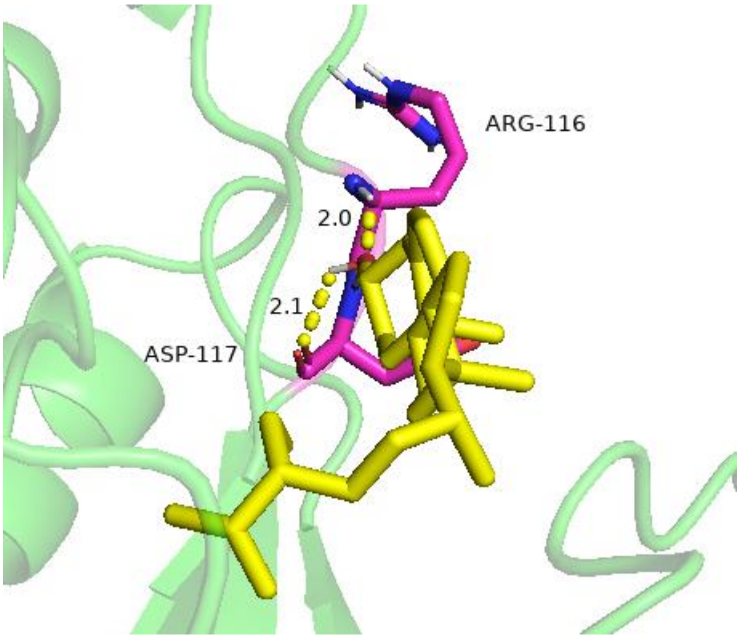

b. AKT1 and lactiflorin

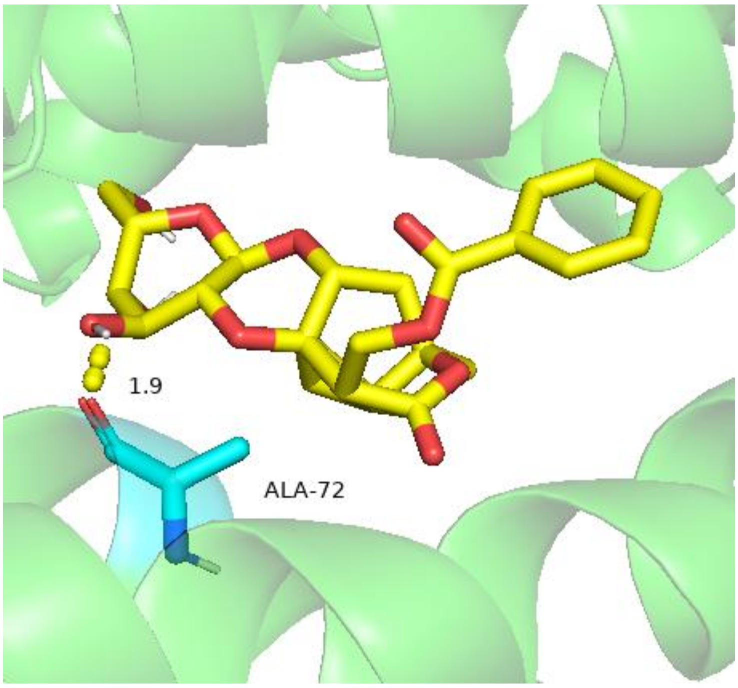

c. AKT1 and dihydroartemisinin

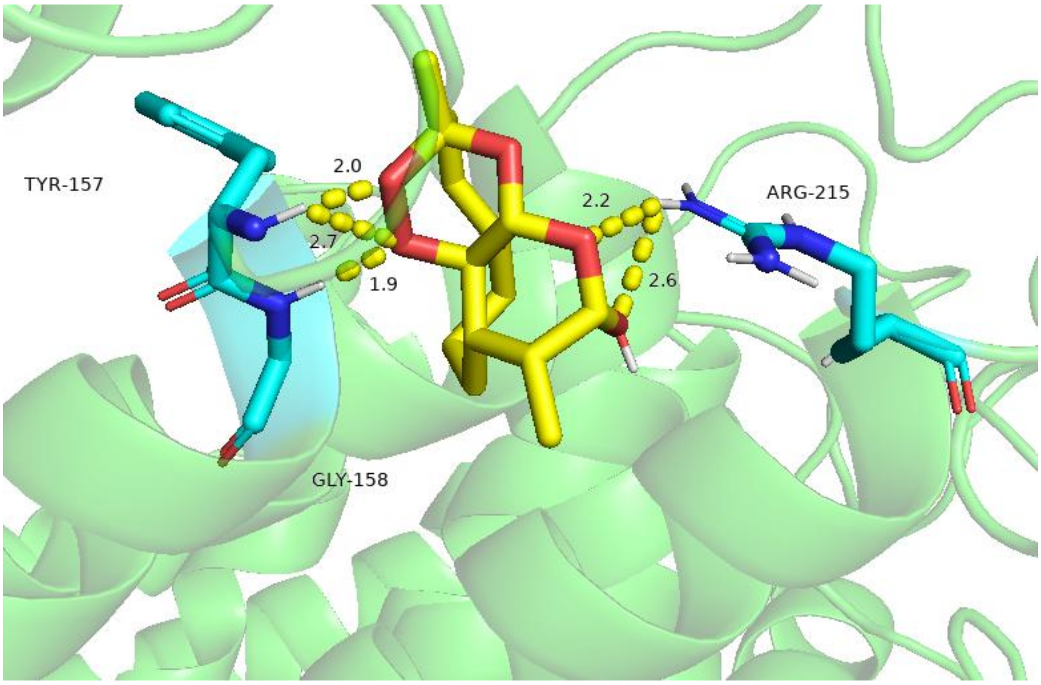

d. MAPK1 and mairin

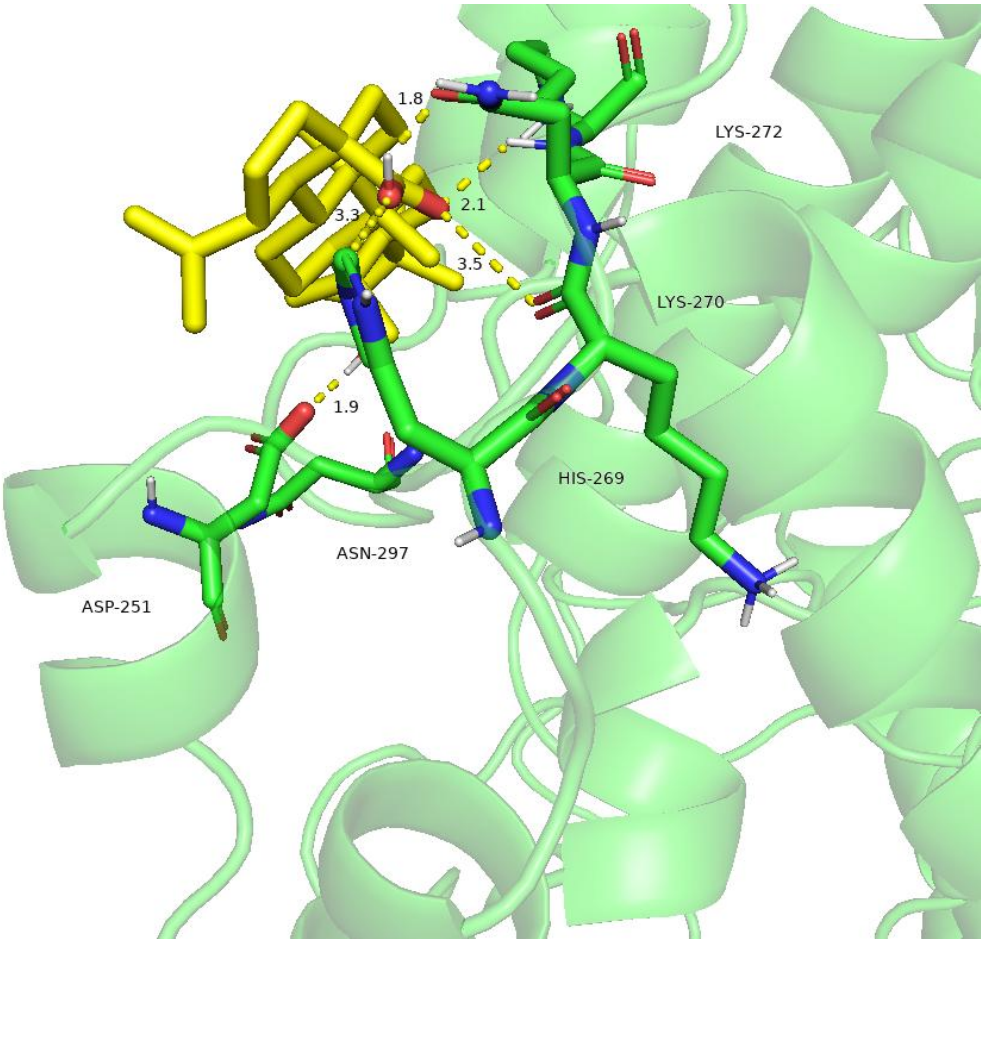

e. RELA and mairin

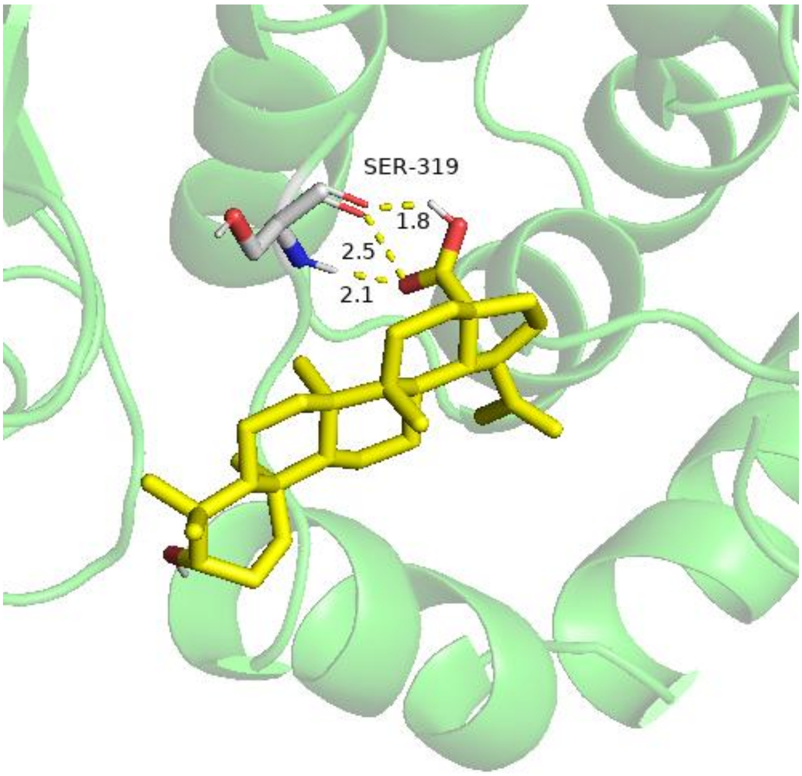

f. AKT1 and mairin

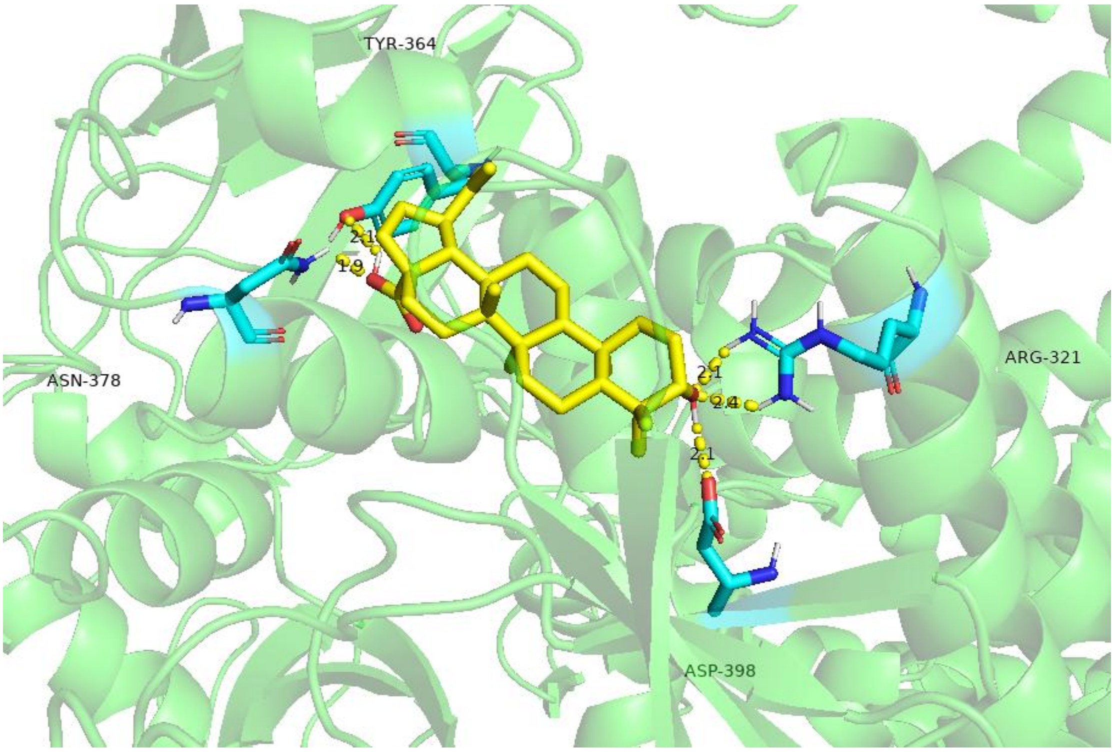

g. RELA and β-sitosterol

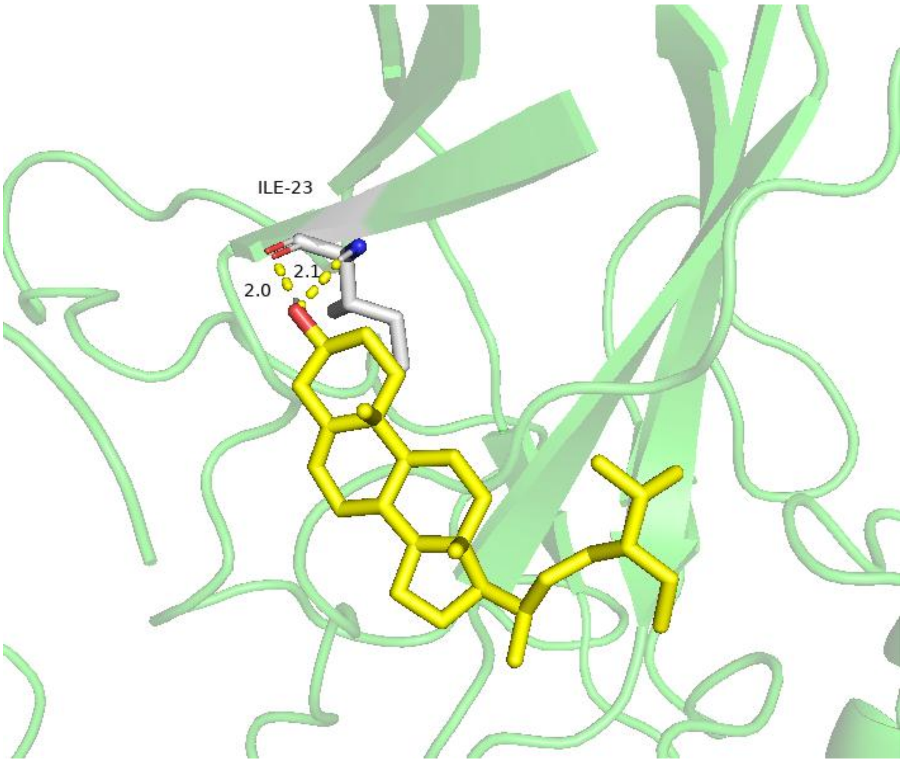

h. MAPK3 and dihydroartemisinin

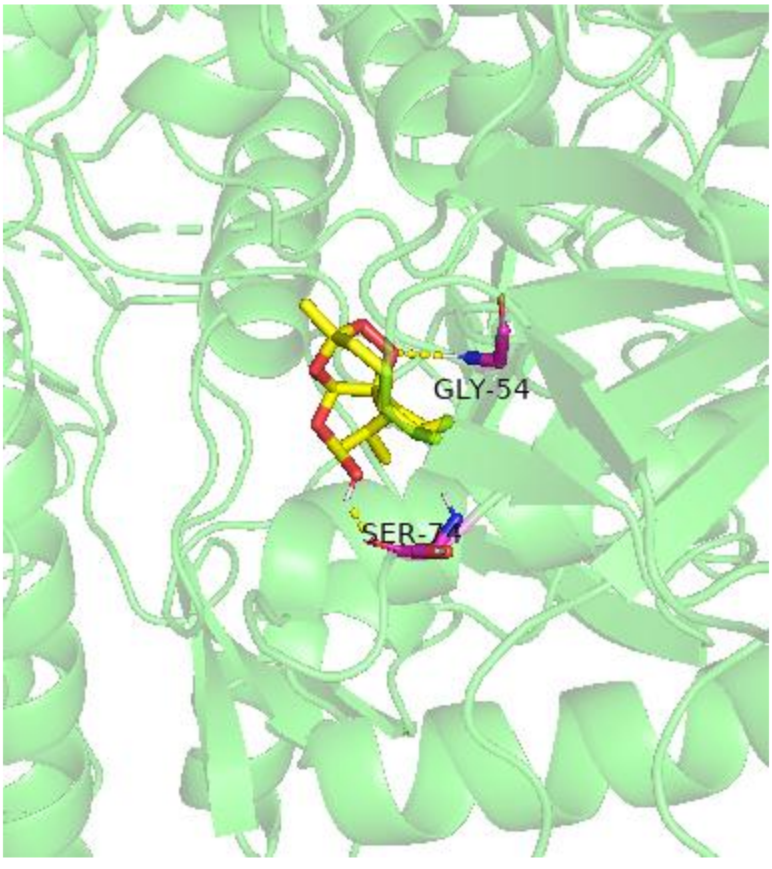

Supplement: Supplementary file 1 [file biomolecules-16-00476-s001.zip › S1. The original images of the molecular docking.pdf]
