# Supplementary figures and images for: A Novel Drug Delivery System for the Treatment of Lupus Nephritis: From Delivery System Design and Optimization to Treatment
Source: Biomolecules. 2026 Mar 23;16(3):476. doi: 10.3390/biom16030476 (PMC13024118; doi:10.3390/biom16030476)

p-ERK

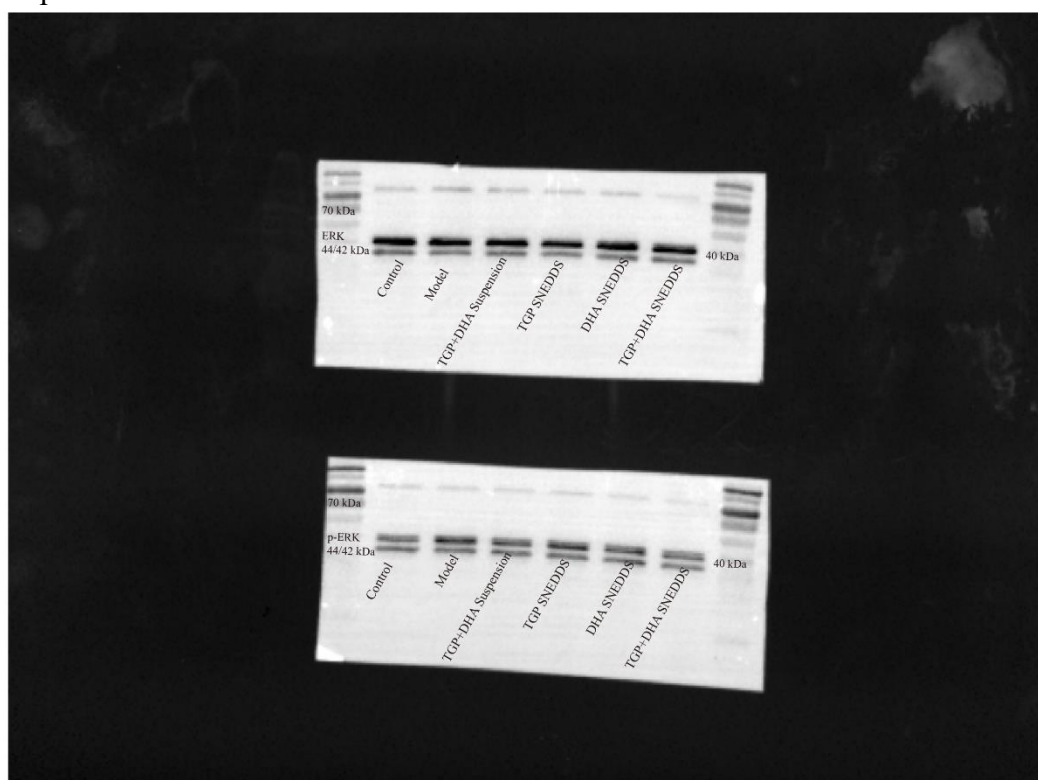

Supplement: Supplementary file 1 [file biomolecules-16-00476-s001.zip › S2. The original images of the Western Blot.pdf]

Model

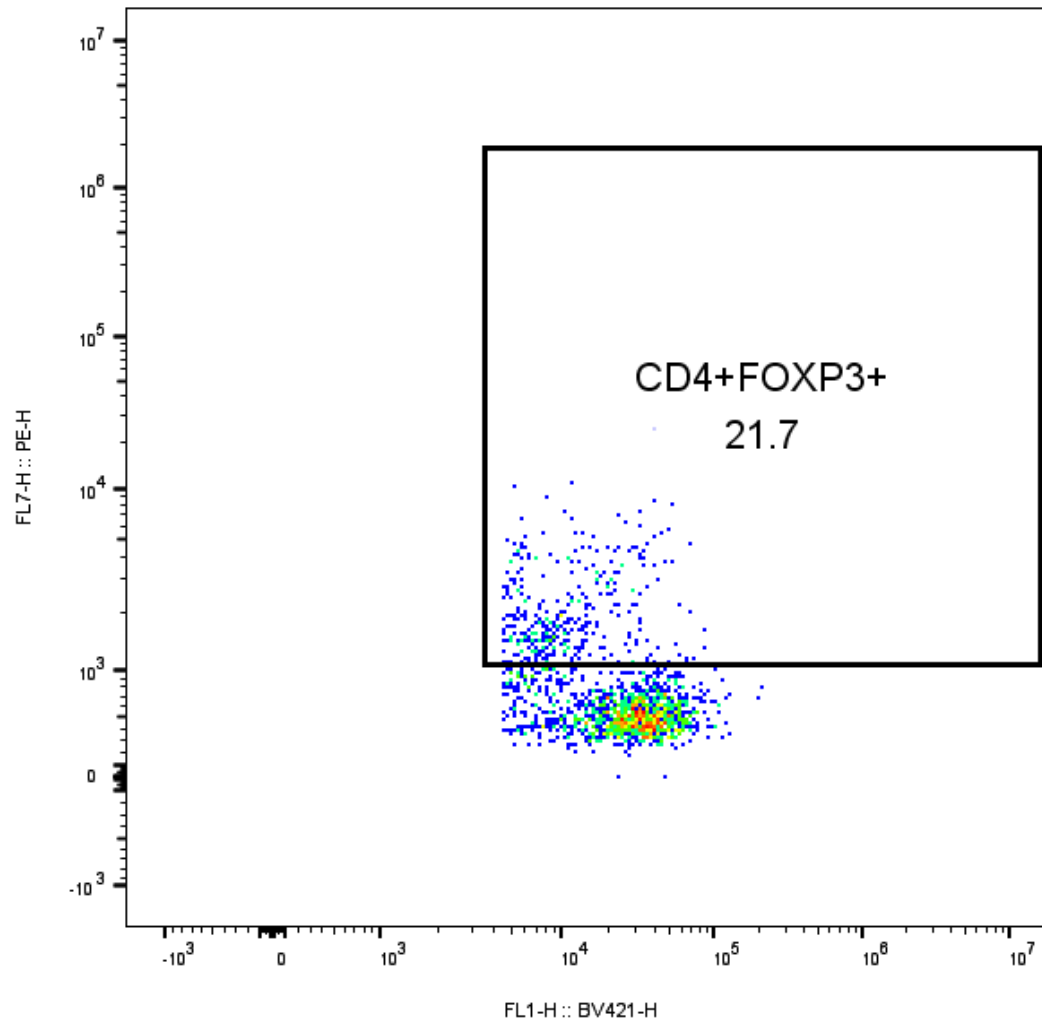

PNS

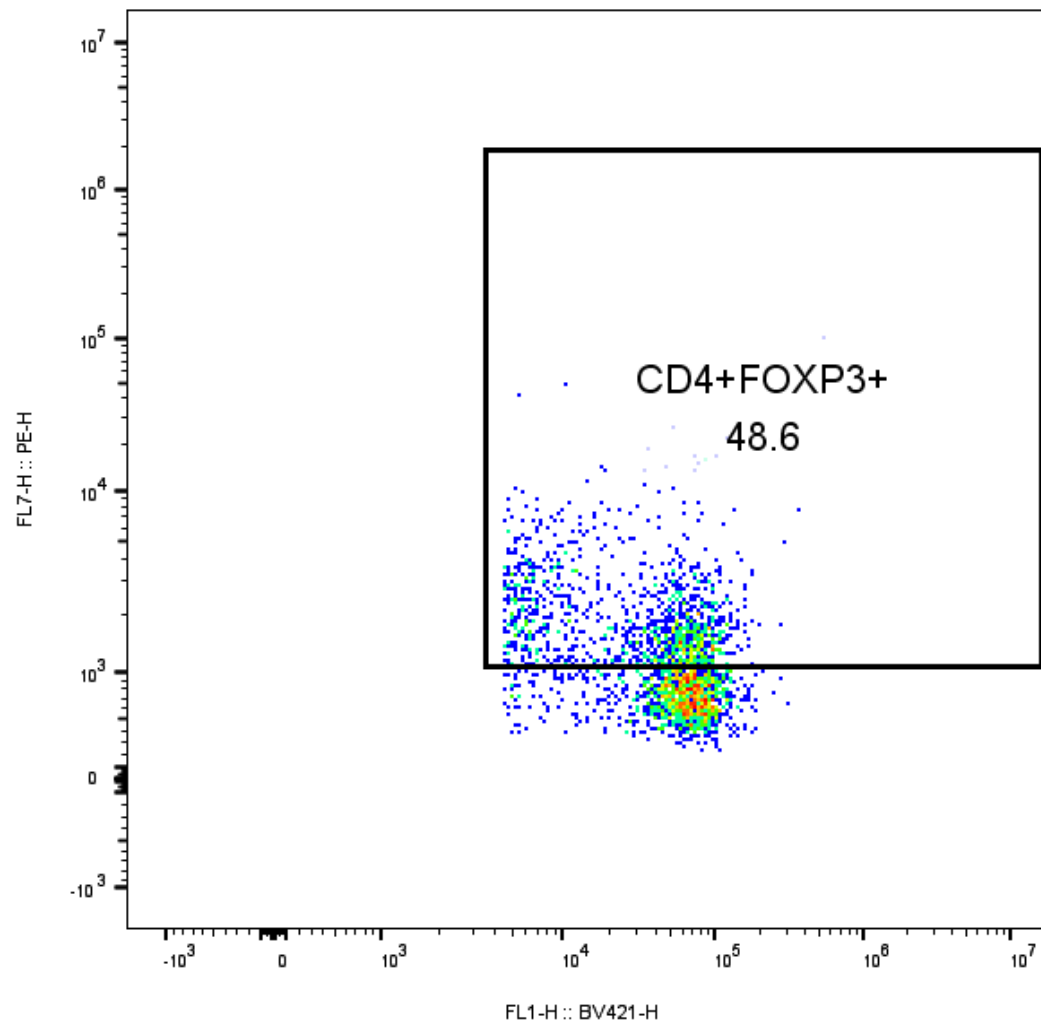

Suspension

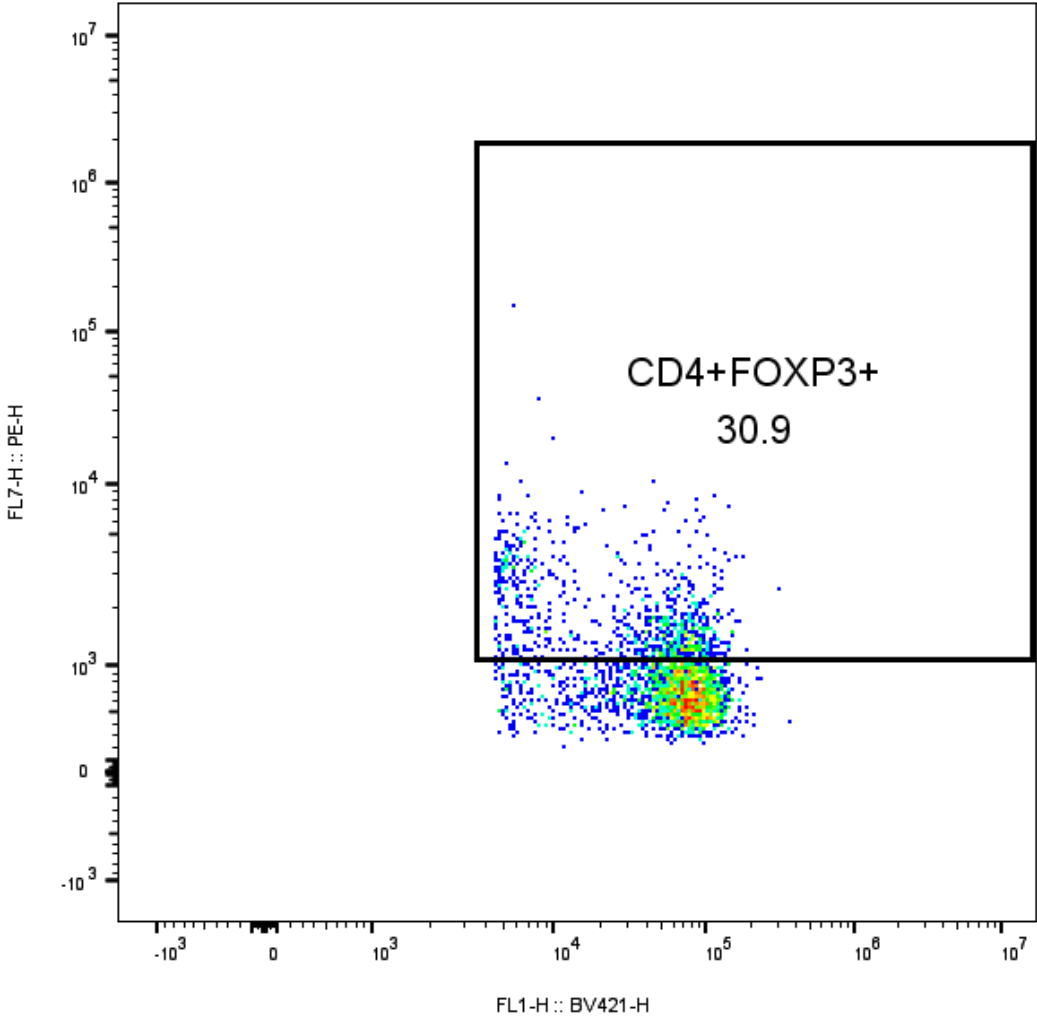

SNEDDS

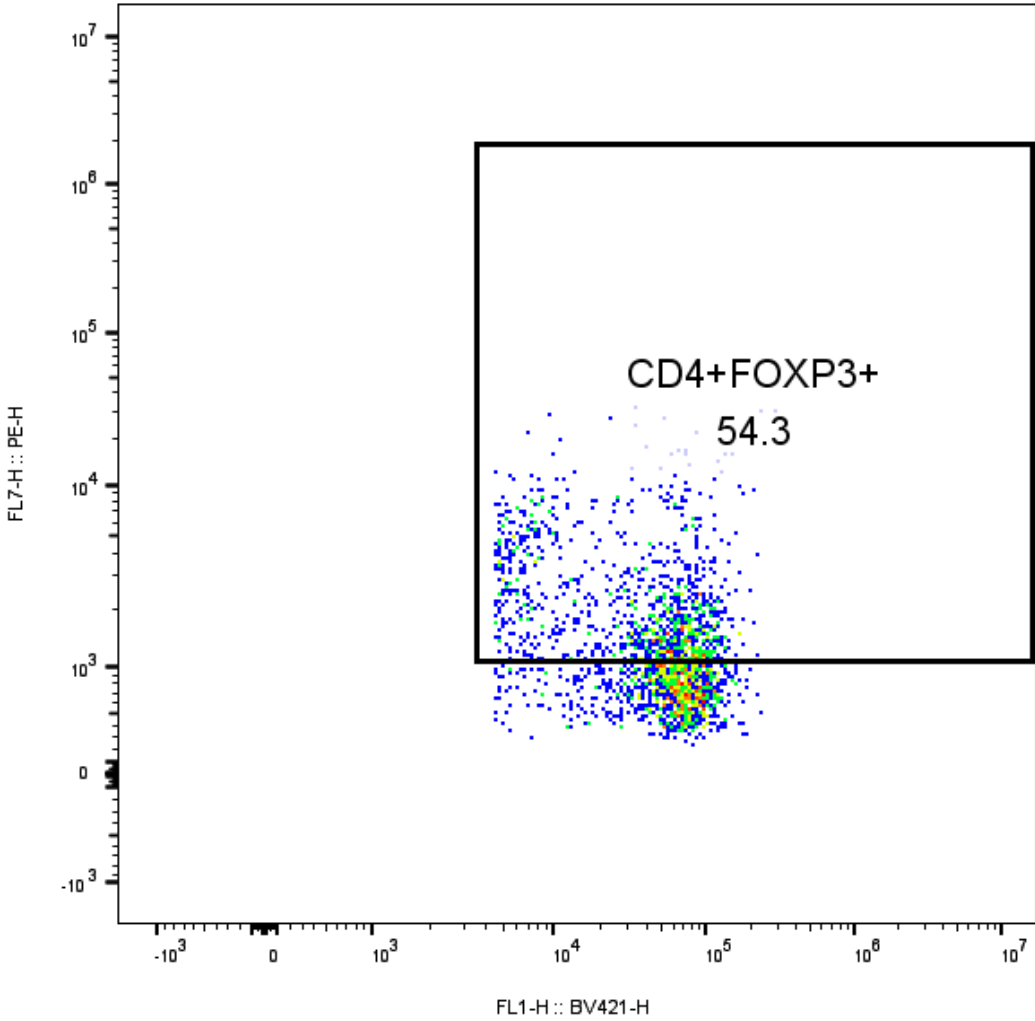

Model

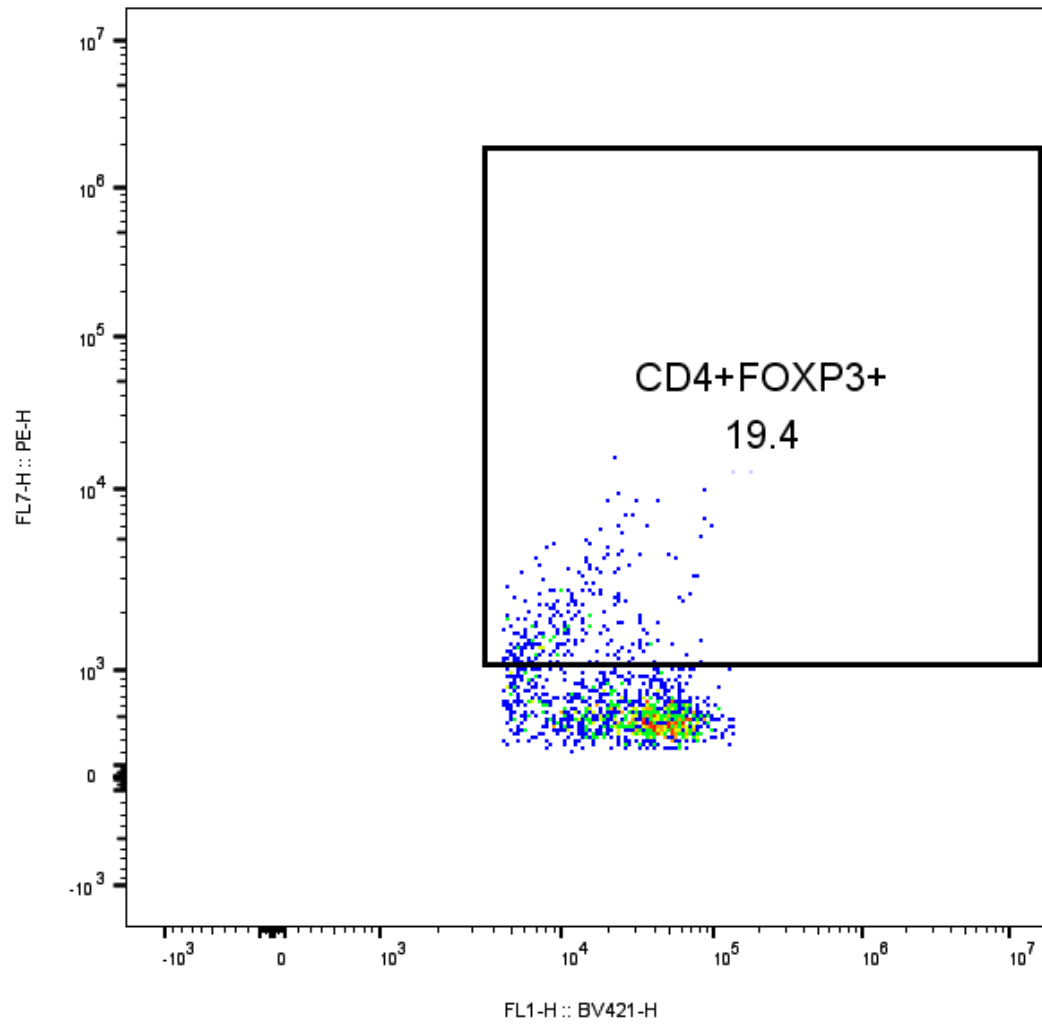

PNS

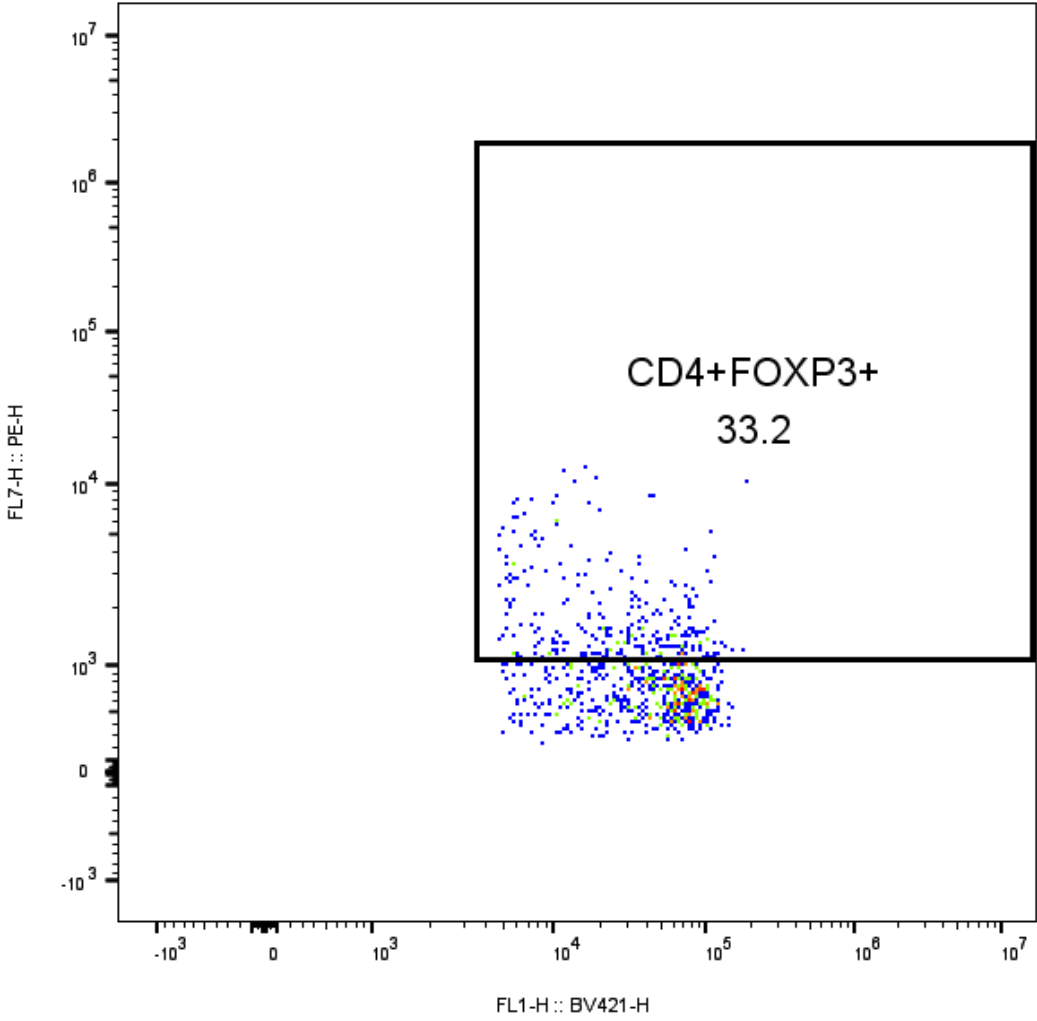

Suspension

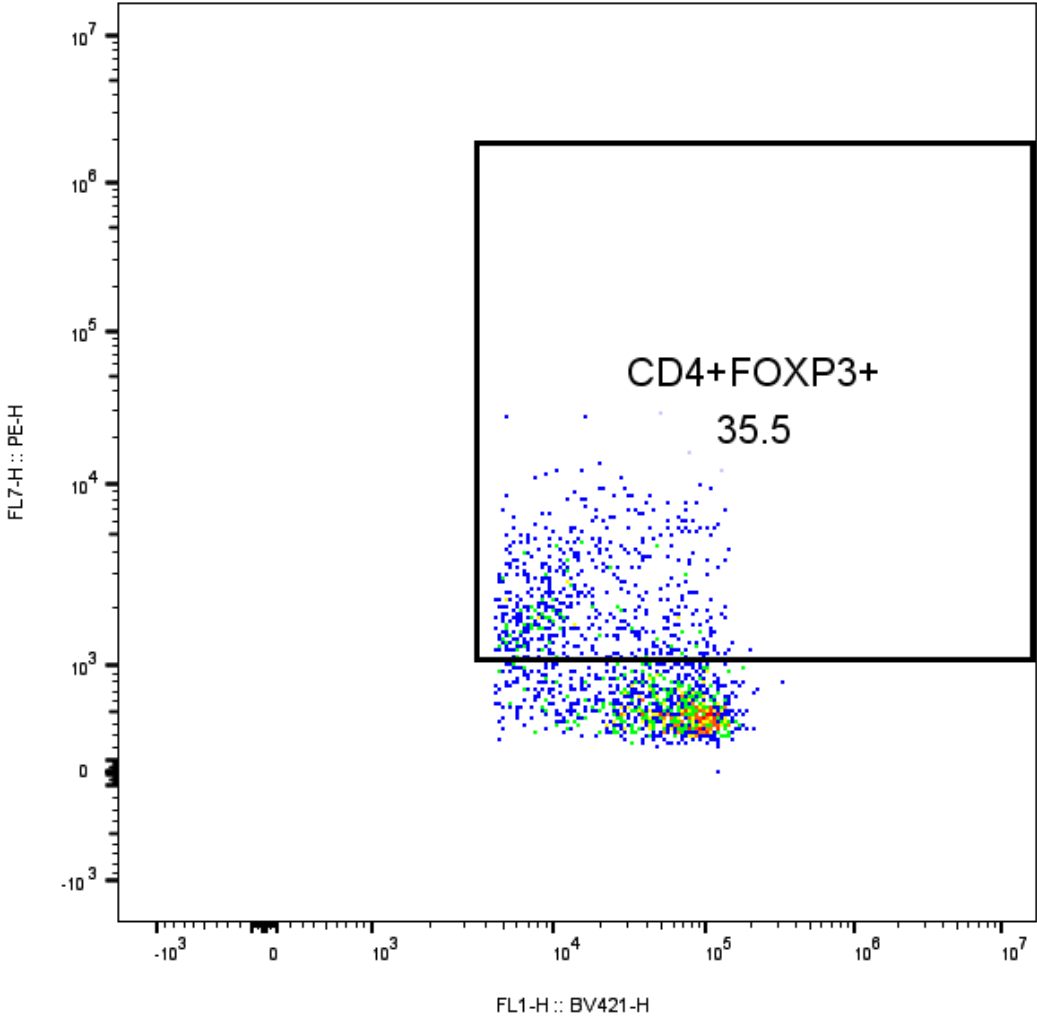

SNEDDS

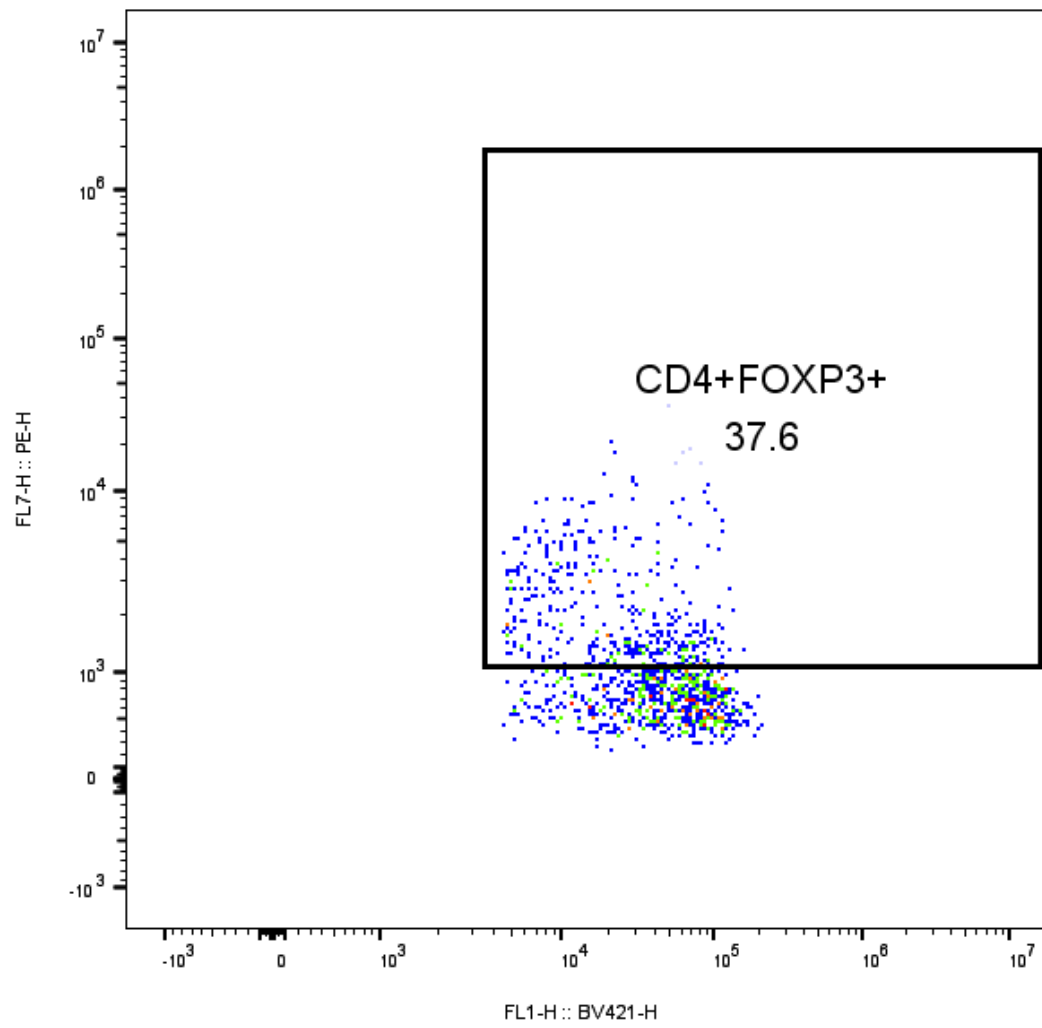

Supplement: Supplementary file 1 [file biomolecules-16-00476-s001.zip › S3. The distribution maps of the flow cytometry detection.pdf]
